# Contrasting nutrient–disease relationships: Potassium gradients in barley leaves have opposite effects on two fungal pathogens with different sensitivities to jasmonic acid

**DOI:** 10.1111/pce.13350

**Published:** 2018-06-29

**Authors:** Jayne L. Davis, Patrick Armengaud, Tony R. Larson, Ian A. Graham, Philip J. White, Adrian C. Newton, Anna Amtmann

**Affiliations:** ^1^ Plant Science Group, Institute for Molecular, Cell and Systems Biology, College of Medical, Veterinary and Life Sciences University of Glasgow Glasgow UK; ^2^ Ecological Sciences The James Hutton Institute Dundee UK; ^3^ Department of Biology, Centre for Novel Agricultural Products University of York York UK; ^4^ Cell and Molecular Genetics The James Hutton Institute Dundee UK

**Keywords:** Blumeria*graminis*, jasmonic acid, potassium, Rhynchosporium*commune*

## Abstract

Understanding the interactions between mineral nutrition and disease is essential for crop management. Our previous studies with Arabidopsis thaliana demonstrated that potassium (K) deprivation induced the biosynthesis of jasmonic acid (JA) and increased the plant's resistance to herbivorous insects. Here, we addressed the question of how tissue K affects the development of fungal pathogens and whether sensitivity of the pathogens to JA could play a role for the K–disease relationship in barley (Hordeum vulgare cv. Optic). We report that K‐deprived barley plants showed increased leaf concentrations of JA and other oxylipins. Furthermore, a natural tip‐to‐base K‐concentration gradient within leaves of K‐sufficient plants was quantitatively mirrored by the transcript levels of JA‐responsive genes. The local leaf tissue K concentrations affected the development of two economically important fungi in opposite ways, showing a positive correlation with powdery mildew (*Blumeria graminis*) and a negative correlation with leaf scald (*Rhynchosporium commune*) disease symptoms. *B. graminis* induced a JA response in the plant and was sensitive to methyl‐JA treatment whereas *R. commune* initiated no JA response and was JA insensitive. Our study challenges the view that high K generally improves plant health and suggests that JA sensitivity of pathogens could be an important factor in determining the exact K–disease relationship.

## Abstract 2

Jasmonic acid (JA) is a plant hormone with important roles in leaf senescence and pathogen defence. Our finding that JA biosynthesis is up‐regulated by low concentrations of potassium (K) in leaves suggests that JA may provide the molecular link between plant K status and fungal disease. This hypothesis was further supported by the finding that disease symptoms on barley leaves were positively correlated with leaf K content for the JA‐sensitive powdery mildew (*Blumeria graminis*) but negatively correlated with leaf K content for the JA‐insensitive leaf scald (*Rhynchosporium commune*).

## 1 INTRODUCTION

Reducing the amount of excess mineral fertilizer applied to crops is an essential step towards sustainable food production (White, Broadley, & Gregory, 2012). It is therefore important to understand how food crops respond to changes in nutrient supply. High‐throughput methods for the analysis of transcripts, metabolites, proteins, and enzyme activities have already provided us with detailed information about the molecular responses of plants to varying nutrient supply under controlled conditions, and about the integration of these responses with plant growth (Amtmann & Armengaud, 2009; Chérel, Lefoulon, Boeglin, & Sentenac, 2014; Sulpice et al., 2009, 2010; Tschoep et al., 2009; Wang & Wu, 2013). In the field, nutrient deficiencies are accompanied by other stress factors, most importantly pathogens and pests. Combating disease in crops is already a major drain on agricultural budgets with expenditure ranking third after that for energy and fertilization (Pimentel, 2005; Savary, Ficke, Aubertot, & Hollier, 2012; Tegtmeier & Duffy, 2004). Thus, a more detailed understanding of the relationships between plant responses to nutritional and biotic stresses is needed for rapid progress towards low‐input agriculture.

Availability of mineral nutrients can affect plant susceptibility to pathogens in a variety of ways (Datnoff & Elmer, 2007; Gupta, Debnath, Sharma, Sharma, & Purohit, 2017; Huber, Römheld, & Weinmann, 2012). Some mineral elements, such as nitrogen and sulphur, are constituents of organic compounds that feed, attract, or deter pathogens, whereas others, such as calcium and silicon, determine the mechanical properties of cell walls and influence physical barriers or palatability (Bloem, Haneklaus, Salac, Wickenhäuser, & Schnug, 2007; Datnoff & Elmer, 2007; Halkier & Gershenzon, 2006; Huber, Römheld, & Weinmann, 2012). Potassium (K) fertilization is generally advertised as improving plant health (Imas & Magen, 2000; Wakeel, Gul, & Zörb, 2016; Wang & Wu, 2013), but a close look at the published studies shows that the effect of K on disease is much less predictable. Evidence from over 2,000 laboratory, glasshouse, and field trials indicates that the effect of K fertilization is most beneficial in ameliorating fungal diseases and pests, whereas less benefit is seen for bacterial and viral infections (Perrenoud, 1990; Prabhu, Fageria, Huber, & Rodrigues, 2007). For all classes of pathogens, some studies report no benefit or even a negative impact of K fertilization. As the mode of pathogenicity does not correlate with taxonomic grouping, this might be expected. A correlation with mode of pathogenicity or trophic state that shows more correspondence with mode of recognition or defence might be more significant in determining infection success (Newton, Fitt, Atkins, Walters, & Daniell, 2010). The exact relationship between K supply and disease incidence and severity depends not only on the specific host–pathogen interaction but also on accompanying mechanistic and environmental factors, but these vary between studies and are often poorly documented. There is no shortage of possible mechanistic links between K deficiency and disease. The “usual suspects” include increased sugar content, lack of stomatal control, decreased turgor, and mechanical stability (Amtmann, Troufflard, & Armengaud, 2008). However, it is important to note that experimental studies proving a relationship or even a correlation between K‐induced physiological changes and disease severity are lacking.

Previous work in our laboratories identified K‐dependent changes in metabolites of arabidopsis (Arabidopsis thaliana [L.] Heynh.), such as increases in reducing sugars and accumulation of glucosinolates, which are potentially of relevance to pathogens and pests in K‐deficient plants (Armengaud et al., 2009; Troufflard et al., 2010). K‐deficient arabidopsis plants were found to have greater expression of genes related to the biosynthesis of the phytohormone jasmonic acid (JA) and of genes related to defence, the latter being dependent on the function of the JA receptor COI1 (Armengaud, Breitling, & Amtmann, 2004, 2010; Yan et al., 2009). *AtLOX2*, encoding lipoxygenase 2, which catalyses the first committed step in JA biosynthesis (Delker et al., 2006; Wasternack & Hause, 2013), responded to low K prior to any visible symptoms (e.g., senescence and growth retardation), demonstrating that the induction of the JA pathway was not a secondary effect of stress symptoms (Troufflard et al., 2010). In agreement with the transcriptional regulation of JA‐biosynthesis genes, levels of JA, as well as its precursors 12‐oxo‐phytodienoic acid (OPDA) and hydroxyl‐12‐oxo‐octadecadienoic acids (HODs), were elevated in K‐deficient plants (Troufflard et al., 2010). Although extensive research on JA signalling has been carried out in dicots such as arabidopsis and tomato (Kazan & Manners, 2008; Pathak, Baunthiyal, Pandey, Pandey, & Kumar, 2017; Wasternack & Hause, 2013; Yan et al., 2016), JA signalling pathways in monocots are relatively unexplored (Ding, Yang, Yang, Cao, & Zhou, 2016; Lyons, Manners, & Kazan, 2013; Shyu & Brutnell, 2015). A number of genes induced in response to JA treatment have been identified in barley, but little is known about their function. They are referred to collectively as jasmonate‐induced proteins (JIPs) and known by their molecular weight (Andresen et al., 1992; Wasternack, Parthier, & Mullet, 1997; Weidhase et al., 1987).

In light of the relationship between low plant K status and JA, it is possible that some of the variations in the effects of K nutrition on plant disease evident in the literature are due to different sensitivities of pathogens to JA. Thus, high concentrations of JA or related oxylipins in K‐deficient plants might positively or negatively modulate plant‐inherent defence responses. It has been proposed that necrotrophic pathogens induce plant defences through JA (Dar, Uddin, Khan, Hakeem, & Jaleel, 2015; Glazebrook, 2005; Kazan & Lyons, 2014; Thaler, Humphrey, & Whiteman, 2012) whereas biotrophic pathogens induce plant defences through the JA antagonist salicylic acid (SA). However, this generalisation does not always hold true. For example, treatment of tomato plants with methyl‐jasmonate (Me‐JA) increased resistance to a range of pathogens with both lifestyles (Thaler, Owen, & Higgins, 2004). The issue is further complicated by a complex cross‐talk between JA and SA signalling pathways; whereas antagonistic interactions prevail in early signalling events, synergistic interactions have been reported for systemic responses (Berens, Berry, Mine, Argueso, & Tsuda, 2017; Devoto & Turner, 2005; Loake & Grant, 2007; Mur, Kenton, Atzorn, Miersch, & Wasternack, 2006; Per et al., 2018; Truman, Bennett, Kubigsteltig, Turnbull, & Grant, 2007; Wasternack & Hause, 2013). Finally, crop varieties display a continuous spectrum of resistance to a given pathogen due to allelic variation in many different genetic loci that determine pathogen recognition and inducible defence responses (Moscou, Lauter, Steffenson, Wise, & Soller, 2011; Piffanelli et al., 2004; Seeholzer et al., 2010; Wise, Lauter, Szabo, & Schweizer, 2009; Zellerhoff et al., 2010). Clearly, the effect of low‐K‐induced up‐regulation of the JA pathway on disease needs to be investigated in individual, well‐defined host–pathogen systems before we can understand (and predict) the effects of K supply on disease incidence.

To test the hypothesis that JA is an important factor for the K–disease relationship in crops, we measured K concentrations in leaves of barley (Hordeum vulgare L. cv. Optic) plants grown under different K regimes and related them to transcript levels of JA‐biosynthesis and JA‐responsive genes, and the development of two fungal pathogens. On the basis of agricultural importance and different lifestyles, we selected the obligate biotroph *Blumeria graminis* f. sp. *hordei* (powdery mildew, *B. graminis*) and the hemi‐biotroph *Rhynchosporium commune* (rhynchosporium, *R. commune*). The UK malting barley variety Optic was selected due to its susceptibility to both fungi. Infection with *B. graminis* initiates no hypersensitive response or lesion formation, thereby allowing the fungus to spread across the leaf and to obtain nutrients from epidermal leaf cells (Glawe, 2008). The life cycle of *R. commune* (scald or leaf blotch) includes an early biotrophic phase during which the fungus grows asymptomatic under the cuticle, and a necrotrophic phase during which conidia are formed normally and necrotic lesions become visible on the leaf surface (Avrova & Knogge, 2012). The results obtained suggest that jasmonate‐signalling links plant K status with disease development.

## 2 MATERIAL AND METHODS

### 2.1 Plant material and growth conditions

Barley (H. vulgare L. cv. Optic) seeds were germinated on water‐saturated paper towels in an environmentally controlled growth chamber with 9‐hr light (270 μmol·m^−2^·s^−1^) at 22°C and 15‐hr dark at 18°C and constant 70% relative humidity. After 4 days, seedlings were transferred to hydroponic solution, supported by corrugated plastic sheets, each holding 60 plants, suspended above 10 L of nutrient solution. The control nutrient solution was composed of (in mm) 1.25 KNO_3_, 0.5 Ca(NO_3_)_2_, 0.5 MgSO_4_, 0.625 KH_2_PO_4_, and 2 NaCl. A solution with no added K (−K) was composed of (in mm) 1.0 Ca(NO_3_)_2_, 0.5 MgSO_4_, 0.625 NaH_2_PO_4_, and 1.375 NaCl. Both media contained the following micronutrients (in μm): 42.5 FeNaEDTA, 0.16 CuSO_4_, 45 H_3_BO_3_, 0.015 (NH_4_)6Mo_7_O_2_, 0.01 CoCl_2_, 0.38 ZnSO_4_, and 1.8 MnSO_4_. The nutrient solution in the plant growth containers was replaced every 7 days. Shoots and roots were harvested at the indicated intervals, weighed, frozen in liquid nitrogen, and stored at −80°C.

### 2.2 Preparation of detached leaf segments

Barley seedlings were grown for 14 days in control or −K solutions. Segments that are 40 mm long were cut from the tip, middle, and base parts of the emerged blade of the second leaf (Supporting Information Figure S1). For subsequent analysis of K content, RNA, or oxylipins, the tissue was frozen immediately after cutting. Treatment of the leaf segments with Me‐JA or fungal pathogens is described below.

### 2.3 Determination of tissue water, K, and oxylipin contents

Approximately 100 mg of frozen shoots, roots, or leaf segments was weighed and freeze‐dried overnight. Water content was determined as the loss of weight by drying and expressed as percentage of fresh weight. To determine K content, freeze‐dried tissue from shoots, roots, or leaf segments was incubated in 2 m HCl (100 μl for 1 mg of dry tissue) at room temperature for 48 hr. Tissue debris was removed by centrifugation, and the extracts were diluted 1:500 in ddH_2_O. K was detected using a flame photometer (Sherwood flame photometer 410). K concentrations in the diluted extracts were determined from a standard curve established with solutions containing 15 to 250 μm KCl in 4 mm HCl. Tissue K concentrations were then calculated by multiplication with the dilution factor and the incubated dry weights. Oxylipins were measured in triplicate 50‐mg samples of lyophilized leaf tissue from leaf segments of plants grown for 14 days in control or −K media (20 plants each). Extraction and liquid chromatography–mass spectrometry analysis were carried out according to previously described procedures (Dave et al., 2011). Initial analysis showed that the variation was too large to resolve differences between leaf segments. Therefore, data from all leaf segments grown in either control or −K media were pooled for statistical analysis.

### 2.4 Measurement of transcript levels using quantitative PCR

Total RNA was extracted from leaf tissue using TRIzol® Reagent (Invitrogen, Cat. 15596‐026) and cDNA prepared using the Superscript III™ Reverse Transcriptase kit (Invitrogen, Cat. 18080–044). A 1/10 dilution of the reverse transcription final reaction was prepared; 1 μl of the dilution was used as template for the qPCR consisting of 0.4 μm of each primer and 1× SYBR Green Master Mix (QuantiTect® SYBR® Green PCR Kit; Qiagen, Cat. 2041453), using a Bio‐Rad Chromo 4 with Opticon Monitor 3 software (Bio‐Rad Laboratories, Inc., California). Serial dilutions of corresponding amplification product were used to monitor the amplification efficiency and to transform threshold cycles into concentrations. The PCR conditions were 15 min at 95°C and then 40 cycles of 15 s at 95°C, 30 s at 58°C, and 30 s at 72°C. Transcript levels were normalized to the expression level of α‐tublin (U40042). Primers were as follows: lipoxygenase 2.A (*HvLOX2*, gene bank AK362687) AGTACCTGGGAGGGATGGAG (forward) and TGGTTTCATGAGCTGGTACG (reverse); allene oxide cyclase (*HvAOC*, gene bank AJ308488) GCTACGAGGCCATCTACAGC (forward) and AAGGGGAAGACGATCTGGTT (reverse); 60‐kDa JIP (*HvJIP60*, gene bank BM815987) CAGCAGCGACTTCATTTACA (forward) and ATGGTGTCGCAGACTATCCT (reverse); α‐tubulin (*Hvα‐TUB*, gene bank U40042) AGTGTCCTGTCCACCCACTC (forward) and AGCATGAAGTGGATCCTTGG (reverse).

### 2.5 Treatment with Me‐JA

For treatment with Me‐JA, the middle segments from the second leaf of 14‐day‐old seedlings grown in control nutrient solution were floated on 45 μm Me‐JA (from 0.1 m stock solution in ethanol) dissolved in water or water with the same final concentration of ethanol (control) and incubated for 24 hr in a lit incubator (LEEC) at 17°C. Detached leaf segments were blotted dry on paper towel and transferred to 0.5% agar/120 mg L^−1^ benzimidazole plates for subsequent inoculation.

### 2.6 Treatment with pathogens

Barley leaf segments were placed on 0.5% agar/120 mg L^−1^ benzimidazole plates (Newton, 1989; Newton, Hackett, & Guy, 1998) and incubated in a lit incubator (LEEC) with continuous light (light intensity 200 μmol·m^−2^·s^−1^ at 17°C) for 24 hr before inoculation with the fungal pathogens. *R. commune* isolate 13‐13 from the culture collection at The James Hutton Institute was grown on CZV8CM agar medium (Newton, Hackett, & Guy, 1998) at 17°C in the dark. The mycelia were scraped from 14‐day‐old cultures using a sterile spatula, transferred to a homogenizer containing sterile water, and homogenized for approximately 30 s. The suspension was filtered through glass wool and resuspended in sterile distilled water at a concentration of 10^6^ spores ml^−1^. The leaf area to be inoculated was brushed gently with a trimmed‐down paint brush to disrupt the cuticle (Newton, Searle, Guy, Hackett, & Cooke, 2001). Ten microlitres of 10^6^ spores ml^−1^ solution was dispensed on to each leaf segment. The plates were returned to the 17°C incubator. The severity of infection was assessed by measuring the length of the lesions (Figure S2A). *B. graminis* f. sp. *hordei* was isolated from infected barley leaves. Spores from individual colonies were used to inoculate detached leaf segments with a paint brush, and the fungus was allowed to grow for approximately 2 weeks. To ensure a pure culture, individual colonies were selected twice more. To inoculate the leaf segments uniformly, an inoculation tower was used (Figure S2B). The plate containing the spores was inverted over a sheet of paper and tapped to dislodge the spores. A cone was formed from the paper, and the spores were blown into the inoculation column. The spores were allowed to settle on the leaf segments for 5 min before the lids were replaced, and plates were returned to the lit incubator at 17°C. The level of infection was assessed by counting the number of visible colonies on each leaf segment (Figure S2C) and dividing by the leaf area (measured from photographs using ImageJ). Noninoculated leaf segments kept in the same conditions as the inoculated leaf segments showed no visible signs of deterioration (Figure S2D).

### 2.7 Statistical analysis

Statistical analysis was performed using analysis of variance with Genstat Version 15.1 and calculation of Pearson correlation between parameters measured over time and across the leaf using Minitab 15 statistical software. Correlation coefficients are shown in Table 1, and *P* values for all correlations tested are given in Supporting Information Table S1.

**Table 1:** Water content (% fresh weight) of barley plants grown in control or K‐free (−K) media

Day^a^	Shoot water content (% FW)		
	Control	−K	*P* value^b^
3	90.6	92.6	0.196
6	91.5	89.5	0.169
9	91.9	90.5	0.342
12	92.7	92.0	0.499
15	91.7	90.3	0.444

Leaf region^c^	Tissue water content (% FW)		
	Control	−K	*P* value^d^
Tip	90.1	86.4	0.043
Middle	91.4	90.7	0.334
Base	92.1	91.5	0.225
*P* value^d^	0.217	0.004	

a After transfer to hydroponics.

b Difference of water content in different media.

c Second leaf, as described in Section 2.

d Difference of water content in different leaf segments.

## 3 RESULTS

### 3.1 Leaves of K‐deprived barley plants reach critically low tissue K concentrations

**Figure 1:**
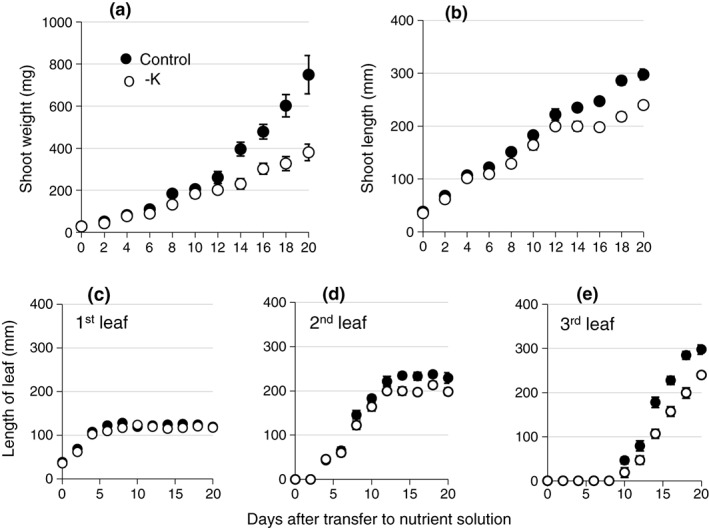
Barley shoot growth in control and −K media. Shoot fresh weight (a), shoot length (b), and length of individual leaves (c–e) of barley plants grown in control (black symbols) or −K (open symbols) media. Five plants were harvested at each time point, and the mean (±*SE*) of three independently grown and treated batches of plants is shown (*n* = 3)

Barley seedlings were transferred to hydroponic culture 4 days after germination and grown on a minimal nutrient solution with either 2 mm K (control) or no K added (−K). No differences in plant size or development were apparent between treatments until 10–12 days after transfer to hydroponics (Figure 1). Subsequently, K‐deprived plants displayed constantly lower shoot fresh weights (Day 12, *n* = 3, *P* = 0.005) and shoot lengths (Day 12, *n* = 3, *P* = 0.013) than control plants (Figure 1a,b). The time point at which K deprivation started to impact visually on growth coincided with the emergence of the third leaf (Figure 1c–e). At this time, seed K reserves for leaf growth will have been exhausted (White & Veneklaas, 2012). The first leaf of K‐deprived plants grew to its full length, and the second leaf showed only a minor reduction in length at the end of its growth period (Figure 1c,d). The third leaf, however, was shorter in K‐deprived plants than in control plants from the beginning of its emergence on Day 10 (Figure 1e). The root fresh weight of K‐deprived plants was also less than that of the control plants grown in full nutrient medium after 10 days (Figure S2A), although the roots were longer than those in control medium (Figure S3B).

**Figure 2:**
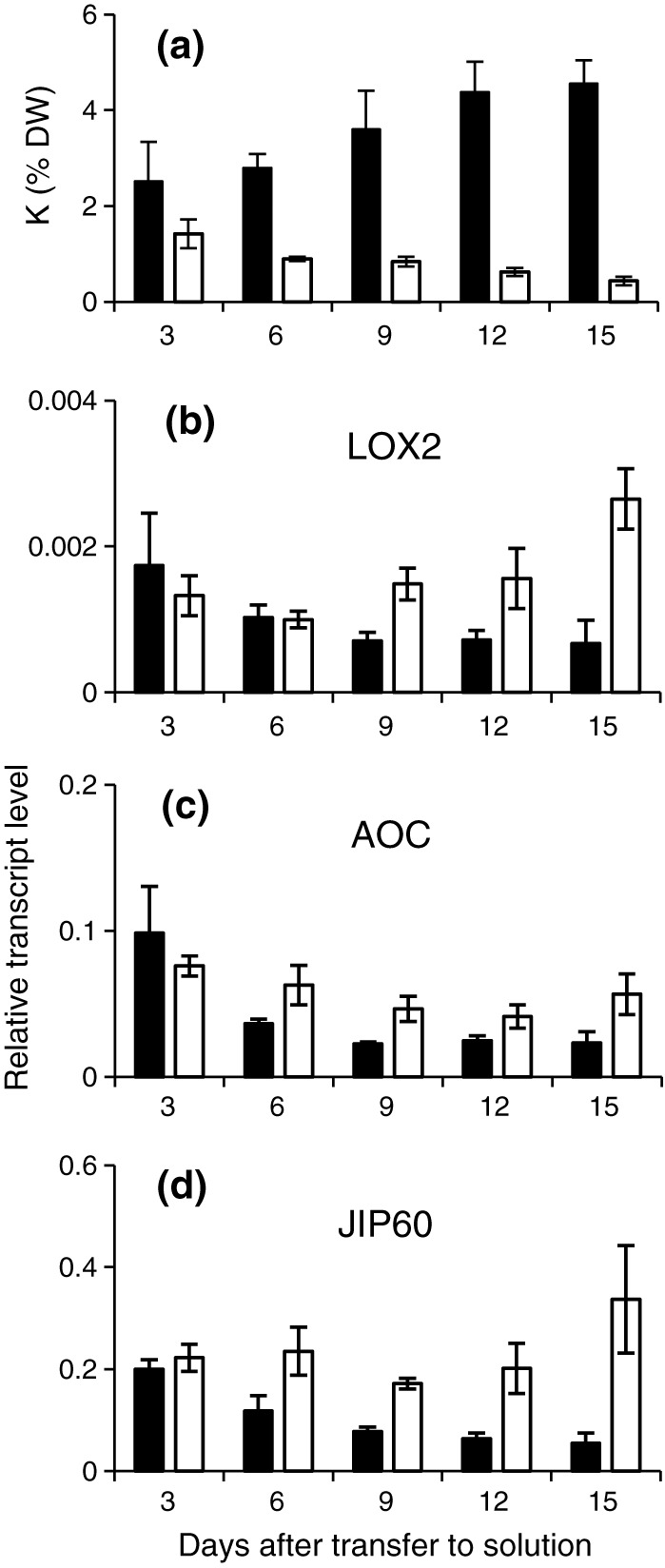
K concentration and JA‐related gene expression in barley grown in control or −K media. Shoot K concentration (a) and relative transcript levels of *HvLOX2* (b), *HvAOC* (c), and *HvJIP60* (d) in barley plants grown in control (black bars) or K‐free (open bars) media. Five plants were pooled for each sample, and the mean (±*SE*) of four independently grown and treated batches of plants is shown. *α‐TUB* was used as reference gene

The K concentration in the medium had an impact on tissue K concentrations, expressed on a dry weight basis, before a difference in fresh weight was apparent (Figure 2, Supporting Information Figure S3). Three days after transfer to hydroponics, the K‐deprived plants already had lower shoot K concentrations than the control plants (1.4% compared with 2.5% dry weight). Over the following 12 days, shoot K concentrations increased in the control plants and decreased in K‐deprived plants (*n* = 3, *P* = 0.012; Figure 2a). The root K concentration in K‐deprived plants was also lower than that in control plants on Day 3 (*n* = 3, *P* = 0.043) and remained constant thereafter while root K concentrations of control plants increased (Supporting Information Figure S3C). On Day 12, the shoot K concentration of K‐deprived plants was only 14% (*n* = 3, *P* = 0.044) and the root K concentration was 22% (*n* = 3, *P* = 0.010) of the shoot K concentration of control plants. From this time point onwards, shoot growth was no longer sustained in K‐deprived plants (Figure 1a,b). Nevertheless, the overall shoot water content was maintained (Table 1).

### 3.2 Leaf K concentration displays a gradient across the emerged blade

**Figure 3:**
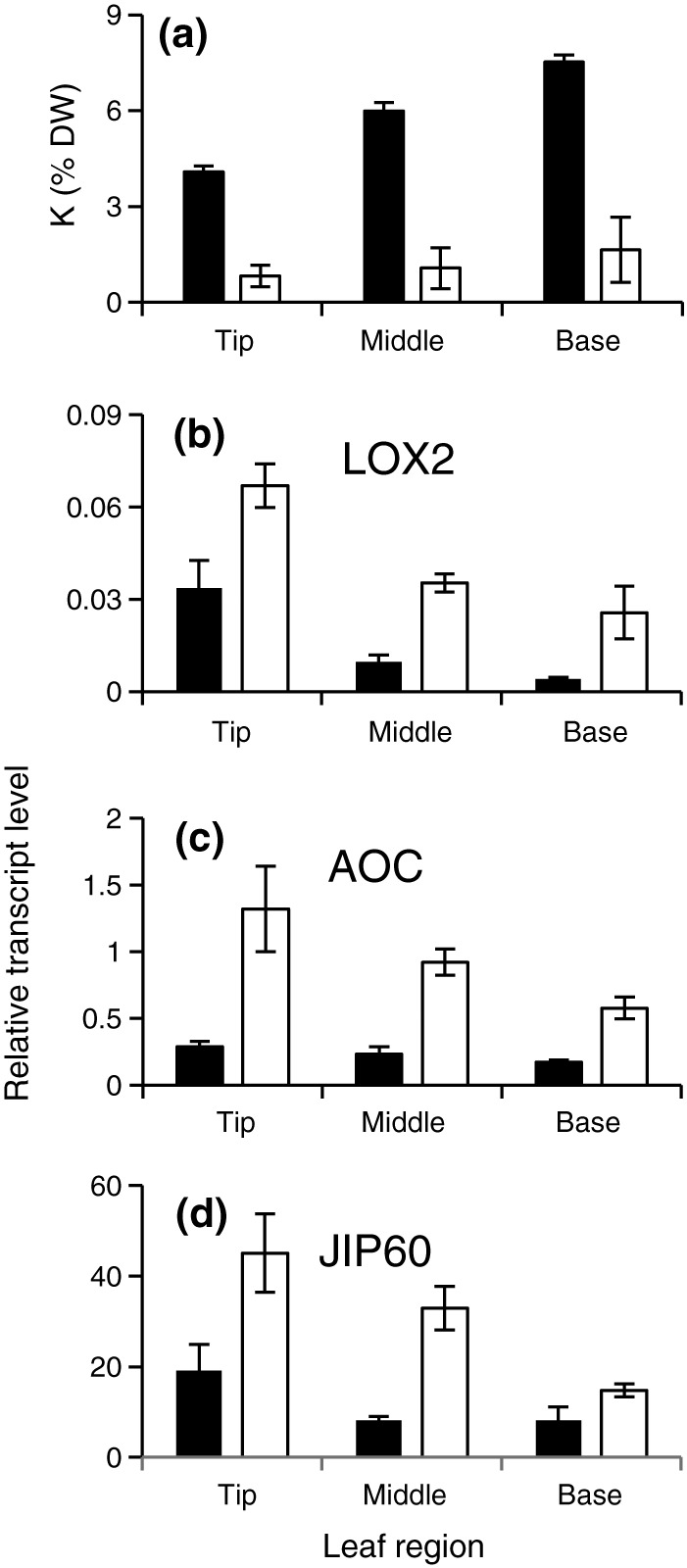
K concentration and transcript levels of JA‐related genes within leaves of barley plants grown in control or −K media. Potassium concentration (a) and relative transcript levels of *HvLOX2* (b), *HvAOC* (c), and *HvJIP60* (d) in different zones of the second leaf of barley plants grown for 14 days in control (black bars) or −K (open bars) media. Corresponding leaf segments from six plants were pooled for each sample, and the mean (±*SE*) of three independently grown and treated batches of plants is shown. *α‐TUB* was used as reference gene

Potassium is mobile in the plant and is preferentially allocated to growing and metabolically active tissues (White & Karley, 2010). Barley leaves are particularly well characterized in this respect; differential allocation of K has been reported in epidermis and mesophyll, in the elongation zone (inside the sheath of the previous leaf) and the emerged leaf blade, and in different sections in the emerged leaf blade (Fricke, Hinde, Leigh, & Tomos, 1995; Fricke, Leigh, & Deri Tomos, 1994a; Karley, Leigh, & Sanders, 2000; Karley & White, 2009; Leigh, Chater, Storey, & Johnston, 1986; Volkov et al., 2009). To investigate spatial differences of tissue K concentrations within the leaf area that is most accessible to airborne pathogens, we measured K concentrations in three zones of the emerged part of the second leaf (base, middle, and tip as shown in Supporting Information Figure S1). In control plants, the K concentration decreased significantly from the base to the tip of the leaf blade (*n* = 3, *P* = 0.012), with the K concentration at the tip being 70% of the K concentration at the base (Figure 3). This is consistent with the observations of Fricke, Leigh, and Deri Tomos (1994b). A decreasing base‐to‐tip leaf K concentration trend was also apparent in K‐deprived plants, although the differences were not statistically significant (Figure 3a). In accordance with the function of K as a major osmoticum, K‐deprived plants showed a significant decrease in water content (expressed as percentage of fresh weight) from the base to the tip of the leaf (*n* = 3, *P* = 0.004; Table 1), and the tip of the leaf was the first part of the plant to show chlorosis and necrosis (Figure S3F). Pearson correlation analysis of the data confirmed a positive correlation between K and water content within the second leaf (*n* = 9, *R* = 0.507, *P* = 0.032; Table 2). In summary, the experimental system allowed us not only to manipulate leaf K concentrations by varying external K supply but also to take advantage of natural differences between local leaf K concentrations within leaves of K‐sufficient plants.

**Table 2:** Pearson correlation coefficients for parameters measured in whole leaves and different leaf zones of plants grown in control and K‐free (−K) media

Parameter measured	Detail	% K	% water	Gene expression						Oxylipins							*Rc* length of lesions			% *Rc* infection			*Bgh* number of colonies			% *Bgh* infection		
				LOX2A	AOS	AOC	JIP23	JIP37	JIP60	JA	OPDA	12‐ODD	9‐HOD	13‐HOD	10‐ODA	OPC‐8	D 6	D 9	D 12	D 6	D 9	D 12	D 6	D 9	D 12	D 6	D 9	D 12
K	% K in DW		0.51	−0.70	−0.78	−0.86	−0.93	−0.90	−0.55	−0.49	−0.51	−0.41	−0.64	−0.68	−0.32	−0.50	−0.76	−0.74	−0.56	−0.73	−0.72	−0.44	0.51	0.39	0.34	0.59	0.67	0.63
Water	% water			−0.74	−0.67	−0.54	−0.60	−0.64	−0.59	−0.21	−0.41	−0.68	−0.41	−0.49	−0.60	−0.39	−0.30	−0.46	−0.69	−0.48	−0.47	−0.55	0.41	0.19	0.13	0.39	0.40	0.51
Gene expression	LOX2A				0.79	0.74	0.71	0.74	0.75	0.44	0.55	0.46	0.64	0.66	0.54	0.56	0.54	0.45	0.52	0.60	0.43	0.42	−0.74	−0.43	−0.41	−0.63	−0.71	−0.70
	AOS					0.87	0.91	0.91	0.85	0.67	0.70	0.67	0.73	0.77	0.24	0.75	0.46	0.59	0.49	0.49	0.57	0.34	−0.67	−0.54	−0.53	−0.66	−0.80	−0.85
	AOC						0.93	0.90	0.69	0.52	0.63	0.46	0.73	0.76	0.22	0.62	0.57	0.69	0.59	0.59	0.70	0.46	−0.57	−0.44	−0.41	−0.60	−0.73	−0.70
	JIP23							0.97	0.70	0.61	0.62	0.59	0.69	0.76	0.25	0.62	0.61	0.73	0.56	0.63	0.71	0.43	−0.55	−0.44	−0.41	−0.58	−0.72	−0.74
	JIP37								0.75	0.60	0.59	0.64	0.70	0.72	0.30	0.59	0.62	0.75	0.62	0.65	0.73	0.52	−0.58	−0.52	−0.51	−0.63	−0.75	−0.79
	JIP60									0.68	0.61	0.66	0.62	0.64	0.25	0.68	0.25	0.32	0.46	0.26	0.30	0.33	−0.75	−0.66	−0.65	−0.63	−0.79	−0.83
Oxylipins	JA										0.28	0.59	0.48	0.41	0.08	0.56	0.16	0.16	0.04	0.07	0.12	−0.13	−0.50	−0.60	−0.56	−0.64	−0.71	−0.67
	OPDA											0.40	0.85	0.92	0.17	0.87	0.15	0.25	0.38	0.19	0.24	0.20	−0.44	−0.24	−0.26	−0.52	−0.50	−0.50
	12‐ODD												0.47	0.53	0.30	0.55	0.08	0.28	0.30	0.13	0.27	0.11	−0.25	−0.37	−0.30	−0.46	−0.40	−0.50
	9‐HOD													0.91	0.20	0.92	0.25	0.36	0.31	0.26	0.35	0.15	−0.54	−0.47	−0.47	−0.80	−0.69	−0.64
	13‐HOD														0.29	0.88	0.35	0.44	0.41	0.36	0.42	0.21	−0.46	−0.32	−0.28	−0.66	−0.59	−0.56
	10‐ODA															0.03	0.36	0.26	0.59	0.38	0.24	0.52	0.00	0.26	0.28	−0.12	0.01	−0.03
	OPC‐8																0.03	0.17	0.15	0.05	0.17	−0.06	−0.57	−0.50	−0.49	−0.74	−0.69	−0.67
*Rc* length of lesions	D 6																	0.82	0.46	0.95	0.78	0.48	−0.35	−0.28	−0.24	−0.36	−0.43	−0.35
	D 9																		0.59	0.86	1.00	0.62	−0.31	−0.32	−0.30	−0.39	−0.48	−0.50
	D 12																			0.53	0.60	0.93	−0.28	−0.04	−0.02	−0.19	−0.26	−0.34
% *Rc* infection	D 6																				0.83	0.56	−0.38	−0.28	−0.24	−0.36	−0.45	−0.39
	D 9																					0.64	−0.29	−0.30	−0.28	−0.37	−0.46	−0.49
	D 12																						−0.26	−0.05	−0.07	−0.09	−0.19	−0.30
*Bgh* number of colonies	D 6																							0.81	0.80	0.73	0.90	0.86
	D 9																								0.97	0.79	0.87	0.81
	D 12																									0.73	0.85	0.82
% *Bgh* infection	D 6																										0.86	0.77
	D 9																											0.95
	D 12																											

*Note*. Significant positive (green) and negative (red) correlations are shaded according to *P* value (<0.05 light, <0.01 medium, <0.005 dark). For exact *P* values, see Supporting Information Table S1. D: day after inoculation.

### 3.3 Transcript levels of JA‐related genes are inversely related to leaf K concentration

Previous research had shown that K deprivation of arabidopsis plants led to increased transcript levels of genes encoding enzymes of jasmonic acid (JA) biosynthesis, such as *AtLOX2*, *AtAOS*, *AtAOC1*, and *AtOPR3* (encoding lipoxygenase, allene oxide synthase, allene oxide cyclase, and OPDA reductase, respectively), as well as well‐known JA targets such as *AtVSP2* (encoding vegetative storage protein; Armengaud, Breitling, & Amtmann, 2004; Armengaud, Breitling, & Amtmann, 2010; Troufflard et al., 2010). To monitor JA response in barley, we used a barley homologue of *AOC1* (AJ308488) and a barley homologue of *LOX2* (gene bank number AK32687). In order to select the most appropriate sequence for *LOX2*, three *LOX2* genes were investigated. All three sequences have higher similarity to the arabidopsis *LOX2* gene than to any other arabidopsis genes encoding lipoxygenases. *LOX2.2* and *LOX2.3* were identified by Bachmann et al. (2002) as *LOX2* genes and shown to be responsive to JA treatment. Our BLAST searches identified a third *LOX2* gene (AK32687, *LOX2.A*). Its closest homologue was the rice *LOX2* gene, and its closest homologue in arabidopsis was *LOX2*. The dendrogram in Supporting Information Figure S4 shows that it is difficult to identify the most likely functional homologue of arabidopsis LOX2 among the three barley genes on the basis of sequence similarity alone. In a preliminary expression analysis with all three genes, we found that *LOX2.A* displayed a more consistent response to −K than did the other *HvLOX2* genes identified, and therefore, we selected it for further study. No *VSP* homologue was found in the available barley nucleotide or protein sequence databases, but a number of Me‐JA‐induced genes (“JA‐induced proteins”, JIPs) have been identified (Andresen et al., 1992; Weidhase et al., 1987). *HvJIP60* (BM815987), used here, encodes a ribosome‐inactivating protein with glycosidase activity (Chaudhry et al., 1994; Dunaeva, Goebel, Wasternack, Parthier, & Goerschen, 1999; Reinbothe et al., 1994). Three barley genes, encoding α‐tubulin (*Hvα‐TUB*, U40042), glyceraldehyde 3‐phosphate dehydrogenase (*HvGAPDH*, M36650), and ubiquitin (*HvUBQ*, M60175), were tested for their suitability as reference genes by determining the variation of Ct values and the frequency distribution of transcript levels obtained by qPCR across a number of different conditions (Supporting Information Figure S5). From this analysis, *Hvα‐TUB* emerged as a robust constitutive reference and was used for normalization of transcript levels.

Transcript levels of *HvLOX2*, *HvAOC*, and *HvJIP60* in shoots of barley plants varied during the experimental period (3–15 days after transfer of the plants to hydroponics), but they were consistently higher in shoots of K‐deprived plants than in shoots of control plants from Day 9 onwards (*n* = 3, LOX2, *P* = 0.027; AOC, *P* = 0.007; JIP60, *P* = 0.002; Figure 2b–d). To establish whether the transcripts responded to tissue K concentration, we analysed different leaf zones of the second leaf. Not only were transcript levels of *HvLOX2*, *HvAOC*, and *HvJIP60* higher in all zones of K‐deprived plants (*P* < 0.001 for all genes) compared with control plants, but they also increased significantly from the base to the tip of the leaf (*n* = 3, *P* = 0.002, *P* = 0.044, and *P* = 0.005, respectively; Figure 3b–d), thus showing the opposite gradient of that observed for tissue K concentration within the leaf (Figure 3a). In summary, the expression of genes in the JA pathway was inversely related to shoot K concentration whether comparisons were made between K‐replete and K‐deprived plants, over the experimental period, or within individual leaves. Indeed, Pearson correlation analysis identified transcript levels of LOX2 (*R* = 0.696, *P* < 0.001), AOC (*R* = 0.731, *P* < 0.001), and JIP60 (*R* = 0.548, *P* = 0.019) as reliable reporters of the overall shoot K concentration, and of local K and water concentrations within the leaf (Table 2).

**Figure 4:**
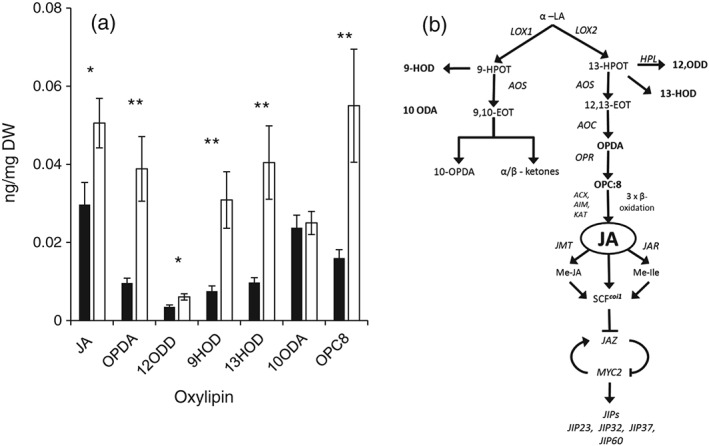
Oxylipin concentrations in leaves of barley plants grown in control or −K media. (a) Oxylipin concentrations in leaves of barley plants grown in control (black bars) or K‐free (open bars) media. Results from tip, middle, and base segments from the emerged blade of the second leaf of 20 plants was pooled for each sample, and means (±*SE*) of three independently grown and treated batches of plants are shown (*n* = 3). (b) Position of oxylipins in the JA‐biosynthesis pathway. Compounds measured are shown in bold; genes encoding enzymes or downstream targets are shown in italics

To test whether the increase in gene expression observed in response to K deficiency was associated with an increase in the concentrations of JA and related compounds, the tissue concentration of several oxylipins was determined (Figure 4) in leaf tissue from plants grown in control or −K media. These included 12‐oxo‐dodecenoic acid (12‐ODD), 13‐hydroxyoctadecatrienoic acid (13‐HOD), 3‐oxo‐2‐(29‐pentenyl)‐cyclopentane‐1‐octanoic (OPC‐8), 12‐oxo‐phytodienoic acid (OPDA), and JA, which are formed in the 13‐LOX pathway. This pathway starts with the conversion of linoleic acid into 13‐hydroperoxy‐9,11,15‐octadecatrienoic acid (13‐HPOT), which is catalysed by LOX2 (Figure 4b, Wasternack & Strnad, 2016). We also measured 9‐hydroxyoctadecatrienoic acid (9‐HOD) and 10‐octadecenoic acid (10‐ODA), which are produced in the 9‐LOX pathway (Figure 4b; Wasternack & Strnad, 2016). The measured oxylipin concentrations were considerably (5–50 times) lower than those previously determined in arabidopsis leaves using the same protocols (Troufflard et al., 2010), and we could not resolve statistically significant differences between the leaf segments (data for all leaf segments are shown in Supporting Information Figure S6). However, clear differences were apparent between control and −K (Figure 4). With the exception of 10‐ODA, all oxylipins measured were found in significantly greater concentrations in the second leaf of the K‐deprived plants than in the second leaf of the control plants (Figure 4a; *n* = 3, *P* < 0.05 for JA and 12‐ODD, *P* < 0.01 for all others). Strong positive correlations were found between the transcript levels of the selected JA marker genes and the concentrations of JA and other oxylipins (Table 2).

### 3.4 Low tissue K has contrasting effects on powdery mildew and rhynchosporium

Typical disease symptoms from *B. graminis* and *R. commune* infection on barley leaves are shown in Supporting Information Figure S2. *B. graminis* colonies form “fluffy” patches (Figure S2B) whereas *R. commune* causes necrotic lesions only visible during this necrotrophic phase (Figure S2A). Development of the fungal pathogens on the leaves was scored by assessing occurrence, number of colonies (*B. graminis*), or size of lesions (*R. commune*) after inoculation of leaf segments from the second leaf, harvested 14 days after transfer of the plants to hydroponics.

**Figure 5:**
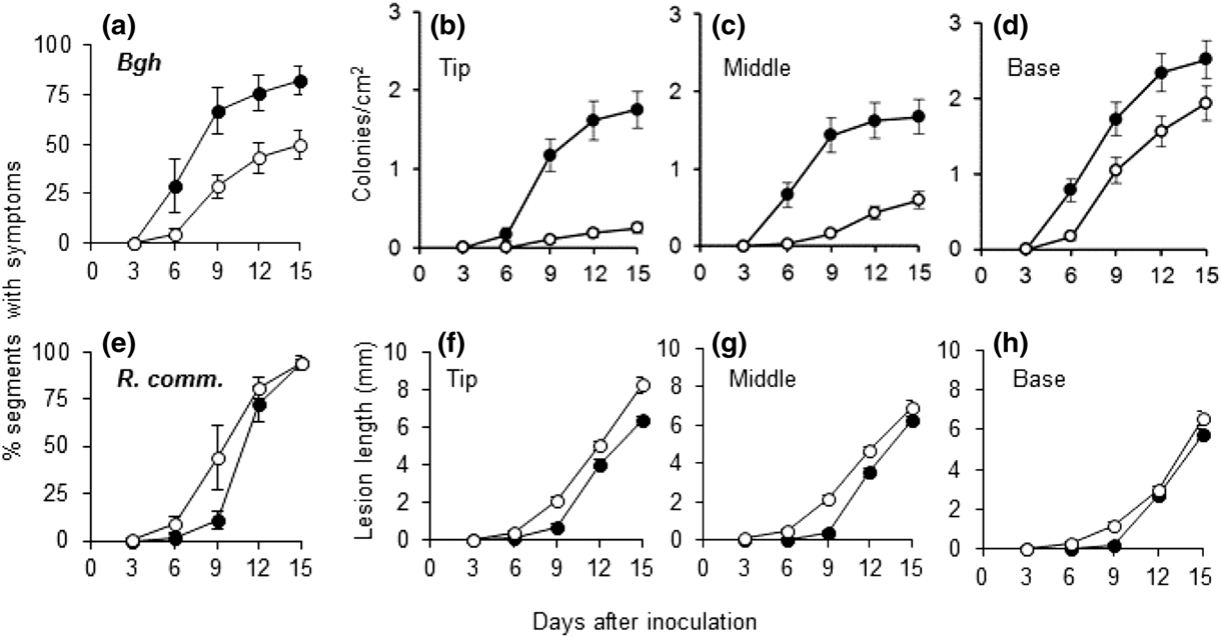
Effect of tissue K concentration on infection by *Blumeria graminis* and *Rhynchosporium commune*. (a, e) Number of barley leaf segments (in % of total number of inoculated leaf segments) showing symptoms after inoculation with *B. graminis* (a) or *R. commune* (e). (b–d) Number of *B. graminis* colonies on segments derived from tip (b), middle (c), or base (d) of the second leaf. (f–g) Length of *R. commune* lesions on segments derived from tip (f), middle (g), or base (h) of the second leaf. Leaf segments were harvested from barley plants grown for 14 days in control (black symbols) or −K (open symbols) media. Means (±*SE*) from three replicate experiments are shown (*n* = 3)

Disease symptoms caused by *B. graminis* were delayed in leaf segments obtained from K‐deprived plants compared with leaf segments from control plants (Figure 5a). In all leaf zones obtained from K‐deprived plants, the number of *B. graminis* colonies was significantly lower than that in leaf zones from control plants (*P* < 0.001; Figure 5b–d). Furthermore, the number of *B. graminis* colonies was always significantly lower at the leaf tip than at the leaf base (*P* < 0.001), for both K‐deprived and control plants. Pearson correlation analysis showed that *B. graminis* infection (percentage of segments inoculated with visible colonies) was positively correlated with the local tissue K concentration measured before inoculation (e.g., *R* = 0.687, *P* = 0.003 for Day 9; Table 2). Thus, a low tissue K concentration in the leaves seems to protect barley against powdery mildew. Correlation analysis also revealed a significant negative correlation between *B. graminis* and transcript levels of JA‐related genes or oxylipin concentrations (Table 2).

Low tissue K concentrations had the opposite effect on disease symptoms caused by *R. commune*. Necrotic lesions appeared earlier in leaf segments obtained from K‐deprived plants than in segments from control plants (Figure 5e), and the individual lesions were significantly larger (*P* < 0.001; Figure 5f–h). In accordance with an effect of local tissue K concentration on *R. commune* infection, lesions were smaller at the base of the leaf than at the tip of the leaf for both control and K‐deprived plants. Pearson correlation analysis showed that the severity of *R. commune* symptoms was directly and negatively correlated with the K concentration measured before inoculation (Table 2). Thus, a low tissue K concentration in barley leaves seems to promote the development of *R. commune*.

### 3.5 B. graminis, but not R. commune, is sensitive to Me‐JA and induces JA‐related genes

**Figure 6:**
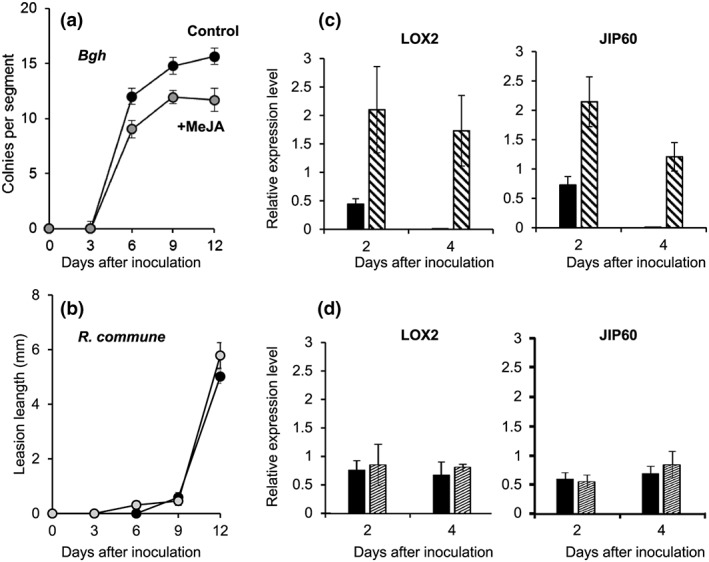
Sensitivity of *Blumeria graminis* and *Rhynchosporium commune* to Me‐JA treatment and inducibility of JA‐related genes. (a, b) Number of *B. graminis* (*Bgh*) colonies (a) and length of *R. commune* lesions (b) on barley leaf segments pretreated for 24 hr with 45 μm Me‐JA (black circles) or water (control, grey circles). (c–f) Relative transcript levels of *HvLOX2* (c, d) and *HvJIP60* (e, f) in uninoculated leaf segments (black bars) or leaf segments inoculated (patterned bars) with *B. graminis* (c, e) or *R. commune* (d, f). *α‐TUB* was used as reference gene. Experiments were performed on middle segments of the second leaves of plants grown for 14 days in control conditions. Means (±*SE*) from three replicate experiments (*n* = 3)

The preceding results suggest that induction of the JA signalling pathway by low K nutritional status may protect barley plants against powdery mildew but not against *R. commune*. This hypothesis is consistent with reports that external application of Me‐JA or other oxylipins to barley inhibited powdery mildew development both locally and systemically (Cowley & Walters, 2005; Schweizer, Gees, & Mosinger, 1993; Walters, Cowley, & Mitchell, 2002) but had variable effects on infection by *R. commune* (Steiner‐Lange et al., 2003; Walters et al., 2014; Weiskorn, Kramer, Ordon, & Friedt, 2002). These previous studies used different barley varieties and growth conditions; therefore, we compared JA sensitivity of the two fungal pathogens in our experimental system directly (Figure 6a,b). Plants were grown in hydroponics with control medium for 14 days, and middle leaf segments were floated on a solution with or without Me‐JA prior to inoculation with the fungi. The Me‐JA treatment reduced the number of *B. graminis* colonies (*n* = 3, *P* < 0.001; Figure 6a) but had little effect on *R. commune* symptoms (Figure 6b). Thus, Me‐JA treatment mimicked the effect of low tissue K concentration on powdery mildew but was ineffective on *R. commune*. Furthermore, transcript levels of *HvLOX2* and *HvJIP60* were increased after inoculation with *B. graminis* (*n* = 3, *P* < 0.001 for both genes) but not after inoculation with *R. commune* (Figure 6c–f). These data suggest that barley uses a JA‐based defence against JA‐sensitive powdery mildew but not against JA‐insensitive rhynchosporium.

In summary, using defined growth and treatment protocols of barley and taking advantage of an inherent K gradient within the emerged blade of the second leaf, we have shown opposite effects of low tissue K concentrations on *B. graminis* and *R. commune* (decreased/increased), different sensitivities of the fungi to JA (sensitive/insensitive), and different inducibility of the JA pathway by the fungi (induced/not induced).

## 4 DISCUSSION

### 4.1 Barley leaves are an excellent system to study nutrient–pathogen interactions

Understanding the interactions between mineral nutrition and disease in plants is essential for good crop management and for making agriculture more sustainable in the future. Molecular plant science has made important contributions to understanding how plants respond to nutritional or biotic stresses, but it is now necessary to (a) design experiments that allow us to assess combined stress and (b) translate knowledge gained in model organisms to crops. In this study, we have done both; using a controlled hydroponics system, we have assessed the effects of plant nutritional status on fungal infection in barley. Measurement of several parameters (ions, transcripts, hormones, and disease symptoms) allowed us to relate these parameters to each other directly. In addition, we have exploited the differential allocation of nutrients within leaves of barley to relate disease symptoms to tissue nutrient concentrations independent of the amount of nutrient supplied in the growth solution. The second leaf was selected for the latter experiments because it grew similarly well in control and K‐deprived plants over most of its growth period but reached critically low K concentrations in its tip towards the end of this time. The experimental system developed here provides a useful tool for studying nutrient–pathogen interactions in barley and other cereal crops.

### 4.2 The K–JA relationship: Possible signals and physiological functions

Previous work by our groups had discovered a strong effect of K deficiency on the JA biosynthesis and signalling pathways in arabidopsis (Armengaud, Breitling, & Amtmann, 2004; Armengaud, Breitling, & Amtmann, 2010; Troufflard et al., 2010). Many of the downstream targets of JA signalling (e.g., production of glucosinolates) are particularly prominent in Brassicaceae, and it was therefore conceivable that the JA response to K deprivation was limited to species of this angiosperm family. The results presented here show that this is not the case. Transcript levels of *HvLOX2* and *HvAOC*, encoding JA‐biosynthetic enzymes that underlie positive feedback regulation by JA in arabidopsis (Delker et al., 2006), and of *HvJIP60*, previously identified in a screen for Me‐JA inducible genes in barley (Andresen et al., 1992; Wasternack, Parthier, & Mullet, 1997; Weidhase et al., 1987), were consistently increased in K‐deprived barley plants (Figure 2). More strikingly, the relative levels of these three transcripts increased from the base to the tip of the emerged blade of the second leaf and thus displayed a gradient that was the inverse of the tissue K concentration gradient, even in plants that were grown in K‐sufficient conditions (Figure 3). We conclude that the expression of the genes is quantitatively determined by variation in tissue K concentration, whether the latter is the result of external supply or of endogenous tissue allocation. At this stage, we cannot distinguish whether the local K signal for JA metabolism is apoplastic or intracellular, and we can only speculate about the downstream events. A number of early signals in wounding and pathogen responses, for example, change in membrane potential, rise of cytoplasmic calcium, and H_2_O_2_ production (Maffei, Mithöfer, & Boland, 2007; Thordal‐Christensen, Zhang, Wei, & Collinge, 1997; Yang, Shah, & Klessig, 1997), also occur in response to reduced apoplastic K (Allen et al., 2001; Amtmann, Troufflard, & Armengaud, 2008; Armengaud et al., 2009; Shin & Schachtman, 2004). However, whether these signals can be quantitative and can persist long enough to explain a continuous dose–response gradient within the leaf is uncertain.

More intriguing is the observation that constitutively high activity of the vacuolar cation channel TPC1 in the arabidopsis *fou2* mutant results in high *LOX2* activities (Bonaventure et al., 2007). The vacuole plays an essential role in cellular K homeostasis because it is used as a reversible K reservoir to maintain stable cytoplasmic K over a wide range of external K concentrations (Carden, Walker, Flowers, & Miller, 2003; Walker, Leigh, & Miller, 1996; White & Karley, 2010). Trans‐tonoplast K fluxes through vacuolar channels will therefore reflect tissue K status in a quantitative manner. Indeed, TPC1 is permeable to K and has been implicated in K homeostasis (Amtmann & Armengaud, 2007; Beyhl et al., 2009; Peiter et al., 2005; Ranf et al., 2007), although it is not clear whether the link is direct (K transport through TPC1) or indirect.

Another good candidate for mediating between cellular K status and defence responses would be calcium. Single‐cell measurements of ion concentrations in different parts of barley leaves have shown a negative correlation between vacuolar concentrations of K and Ca (Fricke, Hinde, Leigh, & Tomos, 1995). It has also been shown before for arabidopsis leaves that a decrease of tissue K under K starvation is compensated by a rise of Ca (Armengaud et al., 2009). Although it is unlikely that a change of the vacuolar Ca concentration directly impacts on the development of fungal pathogens, it could alter the signature of intracellular Ca signals in response to pathogens and thus impact on defence responses. Genetic manipulation of vacuolar K and Ca transporters in barley needs now to be undertaken to investigate whether it is possible to uncouple cellular K and/or Ca homeostasis from JA signalling and whether fluxes of K and/or Ca across the tonoplast underpin the effect of K on pathogen development.

The highest expression of *HvLOX2*, *HvAOC*, and *HvJIP60* was measured in the tips of leaves of K‐deprived plants, which not only had the lowest K concentration but also were the first parts of plants to show chlorosis and a significant drop in water content. It has been shown for arabidopsis that induction of two senescence‐associated genes, At*SAG12* and At*SAG13*, by K deprivation no longer occurred when JA antagonists SA and acetyl SA were applied (Cao, Su, & Fang, 2006). These findings raise the possibility that JA‐related genes inform the plant about local tissue concentrations of the most important cellular osmoticum, K^+^, and induce senescence when tissue K concentration falls below a critical threshold.

### 4.3 What underlies the differential effect of leaf K on *B. graminis* and *R. commune*?

The question of how K deprivation affects the susceptibility of barley to different fungal pathogens was addressed by infecting leaves from control and K‐deprived plants with *B. graminis* and *R. commune*, two economically important pathogens with biotrophic and hemi‐biotrophic (with a necrotrophic phase) lifestyles, respectively. Inoculation with the fungi requires different techniques, which impacts on symptom assessment. An equal number of *B. graminis* spores are blown over the leaf segment, allowing quantification of fungal invasion by counting colonies. By contrast, *R. commune* is point inoculated as a spore suspension, and therefore, all infection sites potentially produce symptoms. Accordingly, the time it takes for visible symptoms to appear and the size of the necrotic lesions formed were scored. In the future, it would be interesting to dissect, at the microscopic level, the effects of tissue K on different phases of fungal invasion and development.

The protocols used here for inoculation and disease scoring followed established techniques in the pathogen field (Newton, 1989), but potential problems for combined nutrient–pathogen studies should be discussed. The extended incubation of the leaf segments did not lead to any visible deterioration of the tissues apart from chlorosis in a small area adjacent to the cut (see uninoculated segments after 15 days on plates shown in Supporting Information Figure S1D). However, it is possible that the segments lose some K during the incubation period. Therefore, our K–disease results strictly relate to the differences of K/JA status before inoculation. Any potential changes occurring in the segments during the incubation period should be monitored in more detail in the future, and controlled plate experiments should be complemented with whole‐plant experiments on soil.

Compared with control plants, K‐deprived plants showed less disease caused by the biotroph *B. graminis* and more by the necrotrophic life stages of *R. commune*. This finding was surprising in the light of the conventional assignment of biotrophic and necrotrophic pathogens to SA and JA‐based defence pathways, respectively. However, it agrees with previous reports of increased resistance against biotrophic pathogens (including powdery mildews) of the arabidopsis mutant *cev1*, which has constitutively high endogenous JA levels (Ellis, Karafyllidis, & Turner, 2002; Ellis & Turner, 2001). External application of jasmonate has also been shown before to reduce *B. graminis* infection in barley both directly and systemically, under controlled conditions (Schweizer, Gees, & Mosinger, 1993; Walters, Cowley, & Mitchell, 2002).

**Figure 7:**
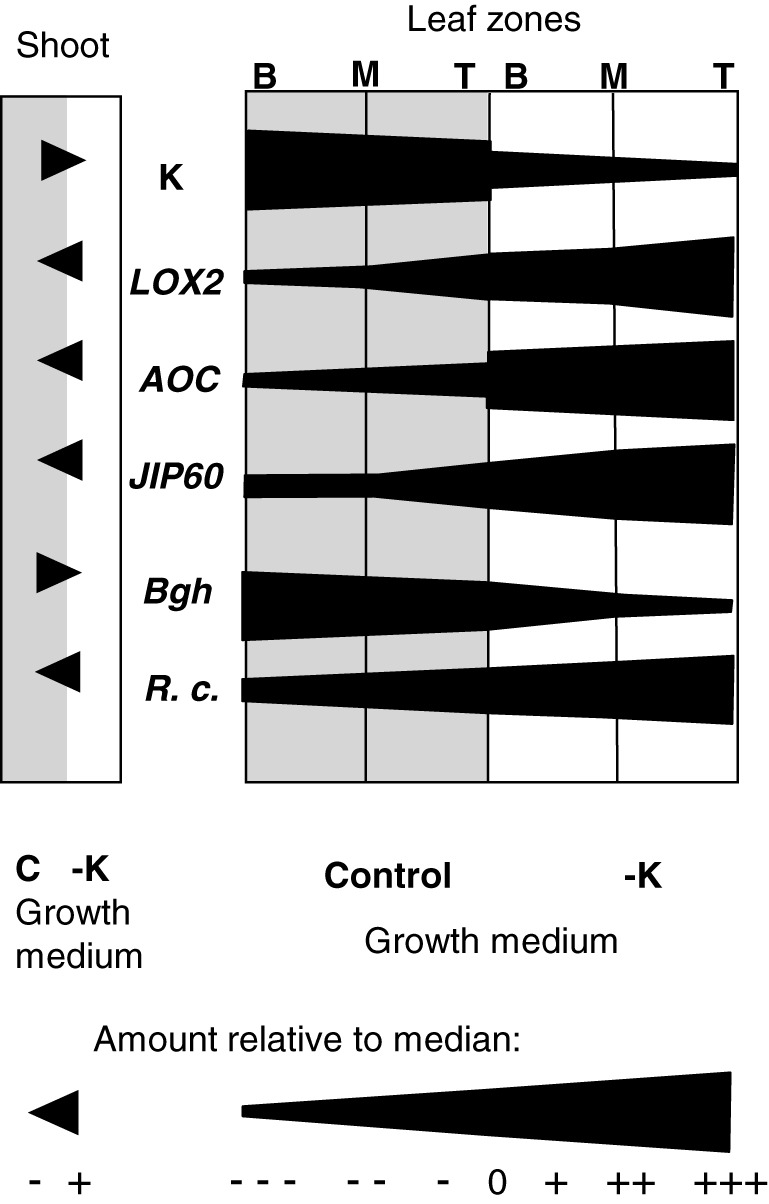
Gradients in tissue K concentration, transcript levels, and disease symptoms across leaf zones and K treatments. Semiquantitative representation of tissue K concentrations (K), transcript levels of JA‐related genes (*HvLOX2*, *HvAOC*, *HvJIP60*) and disease symptoms of *Blumeria graminis* (*Bgh*) and *Rhynchosporium commune* (*R. c*.) in whole shoots (left) as well as base (B), middle (M), and tip (T) regions of the second leaves of plants grown in control (grey background) or −K (white background) media. To build the profiles, measured values were classified into two levels (+, −) for whole shoots or into seven levels for leaf segments, ranging from much lower (−−−) to much higher (+++) than the median (0) across all samples (see scale bar). If amounts differed between adjacent segments, a continuous gradient within the segments was assumed

Further information on the K–disease relationship came from analysing disease symptoms in different leaf regions. Interestingly, occurrence and severity of disease symptoms caused by *B. graminis* and *R. commune* were directly (positively and negatively, respectively) correlated with the local tissue K concentration in leaves even in plants that were K sufficient (control plants). To visualize the leaf profiles of potentially relevant parameters, we assigned a semiquantitative score between −−− (much lower than the median) and +++ (much higher than the median) to the measured absolute values, and we plotted this score against the leaf zones for both K‐replete and K‐deprived plants. As can be seen in Figure 7, *R. commune* and *B. graminis* symptoms display almost continuous gradients across zones and treatments as do tissue K concentrations and transcript levels of *HvLOX2*, *HvAOC*, and *HvJIP60*.

Promotion of *B. graminis* by increasing tissue K concentration meant that this biotroph developed better in K‐rich tissues, particularly at the base of the emerged leaf blade of K‐replete plants. Although this could be due to a direct beneficial role of K as an essential nutrient, it is difficult to conceive that the small differences of K concentration found in K‐replete plants would cause nutritional deficiency in the fungus. It is more likely that the increased JA level in low‐K tissues leads to enhanced plant defence, preventing successful development of *B. graminis*. The opposite effect of K on the JA‐insensitive fungus *R. commune* (inhibition by high tissue K concentrations) is in line with the general view that K protects plants against disease, but it still requires identification of the underlying mechanism(s). It has been reported that *R. commune* infection leads to increased transpiration and K accumulation around stomata (Ayres & Owen, 1971), indicating that stomatal function is modulated either as part of the fungal infection strategy or as a downstream effect. Lowering K may interact with this process and facilitate infection.

The results from this study strongly motivate a new hypothesis that links the effect of tissue K on disease development with the sensitivity of the pathogen to plant JA signalling, but alternative explanations are still possible and should be examined in more targeted studies. For example, K starvation might increase not only vacuolar but also apoplastic Ca, leading to increased rigidity of cell wall and membranes, which in turn could differentially inhibit pathogens depending on their infection paths. Furthermore, the allocation of K and Ca into individual cell types (Fricke, Hinde, Leigh, & Tomos, 1995) could change under K starvation, which again might differentially affect pathogens with specific invasion patterns. With reported effects of JA on ion fluxes being taken into account (Evans, Gottlieb, & Bach, 2003; Yan et al., 2015), it is also possible that an initial rise in JA leads to redistribution of K and/or Ca between cellular compartments and cell types. Monitoring ion concentrations and pathogen development at a much higher spatial resolution would be a good way forward to test these hypotheses. The experimental protocols developed here to score K–disease interaction provide a basis for such studies.

### 4.4 A working model for the K–JA–disease interaction

The results from this study can be summarized in a simple working model (Figure S7) in which a low K concentration in leaf tissue induces JA signalling, which in turn enhances the inducible defence response of the plant against *B. graminis*. In this case, the effect seems to be strong enough to overcome any other effects of low K status that may increase plant susceptibility. By contrast, *R. commune* does not induce a JA‐based defence response, and this pathogen is not sensitive to JA. Induction of JA signalling by low K has therefore no consequence on pathogen development. The observed effect of K on *R. commune* is in accordance with the conventional view that K deficiency promotes disease, but the exact cause still remains to be identified. Our finding that the effect is local and continuous over a range of K tissue concentrations narrows the spectrum of potential causes. For example, levels of sugars increased in −K conditions but were not correlated with K concentrations in the leaf segments (Figure S8).

Interestingly, it has been reported that soil‐grown barley plants exposed to a combination of elicitors (including *cis*‐jasmonate) after preinfection with *R. commune* down‐regulate *LOX2* (although in this case the transcript measured differed from the one assessed here; Walters, Paterson, Sablou, & Walsh, 2011). This raises the possibility that *R. commune* infection may cancel the protective effect of low K on *B. graminis*, observed here. Future experiments should assess the effect of K on simultaneous or successive infection by both pathogens. Depending on which pathogen is more damaging, there might be scope for fine‐tuning K fertilizer applications. Furthermore, the observed differential development of the two fungi in different parts of the leaf could open the possibility of a more targeted application of fungicides.

## Supporting information

Figure S1. Barley leaves and leaf zones. A: Numbering of leaves. B: Leaf segments of second leaf harvested for the analysis
Figure S2. Pathogen symptoms and inoculation procedure. A: Lesions caused by *R. commune* 12 days after inoculation. B: Colonies of *B. graminis* 12 days after inoculation. C: Setup for applying *B. graminis* spores to leaf segments. D: Appearance of un‐inoculated leaf segments after15 days on the inoculation plates.
Figure 3S. Growth and K content of barley roots in control and low‐K conditions. Length (A), fresh weight (B) and K concentration (C) of roots from barley plants grown in control (black symbols) or ‐K (open symbols) media. Five plants were harvested for each time point, the means (± SE) from three independently grown and treated plant batches are shown (n=3). Pictures of representative roots and shoots 20 days after K deprivation are shown in D, E, and F.
Figure S4: Dendrogram showing the relationship between LOX2 gene sequences. Locus identifiers are shown in brackets and bootstrap values in italics.
Figure S5. Identification of suitable constitutive reference gene. A: Variation across samples of transcript levels determined from Ct values in qPCR for UBQ, GAPDH and α‐TUB. B, C: Frequency distribution of transcript levels obtained in qPCR for GAPDH (B) and α‐TUB (C). RNA was prepared from barley plants grown in full nutrient control and low‐K nutrient solution for 3, 6, 9, 12 and 15 days, and transcript levels were normalised to day‐3 control.
Figure S6: Oxylipin concentrations of leaf segments Oxylipin concentrations in leaf segments from barley plants grown in control (black bars) or ‐K (open bars) media. Leaf tissue of tip (A), middle (B) and base (C) segments from the emerged blade of the second leaf of 20 plants was pooled for each sample, and means (± SE) of three independently grown and treated batches of plants are shown. For abbreviations of oxylipin names, see text. ‘Oxy’ is the sum of all measured oxylipins.
Figure S7 Current working model for K‐pathogen relationship. Low tissue K concentrations induce JA‐biosynthesis and signalling (black arrow, data in Figs. 2 and 3) with different consequences for *B. graminis* (A) and *R. commune*(B). A: JA is part of the defence of barley against *B. graminis*. It is induced upon *B. graminis* infection and it inhibits the fungus (data in Fig. 6). JA‐induction by low tissue K therefore protects barley against *B. graminis* (data in Fig. 5). Other physiological factors accompanying low K may weaken the plant's resistance against *B. graminis* (white arrow, literature) but the protective effect of JA outweighs these factors. B: JA is not induced by *R. commune*, and *R. commune* is insensitive to JA (data in Fig.6). The low‐K induced rise of JA has therefore no effect on *R.commune*. Enhanced disease
Figure S8. Sugar concentrations in barley shoots and leaf segments. Concentrations of glucose, fructose and sucrose in whole shoots (A) and in different zones of the second leaf (B) of barley plants grown in control (black symbols) or ‐K (open symbols) media. For A, five plants were pooled for each sample. For B, corresponding leaf segments were pooled from six plants. Means (± SE) of three independently grown and treated plant batches are shown (n=3) development in tissues with low K (white arrow, data in Fig. 2 and 3) is not counteracted by JA.

Data S1 Supporting information item

## References

[pce13350-bib-0001] Allen, G. J. , Chu, S. P. , Harrington, C. L. , Schumacher, K. , Hoffmann, T. , Tang, Y. Y. , … Schroeder, J. I. (2001). A defined range of guard cell calcium oscillation parameters encodes stomatal movements. Nature, 411, 1053–1057. 11429606 10.1038/35082575

[pce13350-bib-0002] Amtmann, A. , & Armengaud, P. (2007). The role of calcium sensor‐interacting protein kinases in plant adaptation to potassium‐deficiency: New answers to old questions. Cell Research, 17, 483–485. 17568763 10.1038/cr.2007.49

[pce13350-bib-0003] Amtmann, A. , & Armengaud, P. (2009). Effects of N, P, K and S on metabolism: New knowledge gained from multi‐level analysis. Current Opinion in Plant Biology, 12, 275–283. 19493694 10.1016/j.pbi.2009.04.014

[pce13350-bib-0004] Amtmann, A. , Troufflard, S. , & Armengaud, P. (2008). The effect of potassium nutrition on pest and disease resistance in plants. Physiologia Plantarum, 133, 682–691. 18331404 10.1111/j.1399-3054.2008.01075.x

[pce13350-bib-0005] Andresen, I. , Becker, W. , Schlüter, K. , Burges, J. , Parthier, B. , & Apel, K. (1992). The identification of leaf thionin as one of the main jasmonate‐induced proteins of barley (*Hordeum vulgare*). Plant Molecular Biology, 19, 193–204. 1377959 10.1007/BF00027341

[pce13350-bib-0006] Armengaud, P. , Breitling, R. , & Amtmann, A. (2004). The potassium‐dependent transcriptome of arabidopsis reveals a prominent role of jasmonic acid in nutrient signaling. Plant Physiology, 136, 2556–2576. 15347784 10.1104/pp.104.046482 PMC523322

[pce13350-bib-0007] Armengaud, P. , Breitling, R. , & Amtmann, A. (2010). Coronatine‐insensitive 1 (COI1) mediates transcriptional responses of *Arabidopsis thaliana* to external potassium supply. Molecular Plant, 3, 390–405. 20339157 10.1093/mp/ssq012 PMC2845782

[pce13350-bib-0008] Armengaud, P. , Sulpice, R. , Miller, A. J. , Stitt, M. , Amtmann, A. , & Gibon, Y. (2009). Multilevel analysis of primary metabolism provides new insights into the role of potassium nutrition for glycolysis and nitrogen assimilation in arabidopsis roots. Plant Physiology, 150, 772–785. 19346439 10.1104/pp.108.133629 PMC2689955

[pce13350-bib-0009] Avrova, A. , & Knogge, W. (2012). *Rhynchosporium commune*: A persistent threat to barley cultivation. Molecular Plant Pathology, 13, 986–997. 22738626 10.1111/j.1364-3703.2012.00811.x PMC6638709

[pce13350-bib-0010] Ayres, P. G. , & Owen, H. (1971). Resistance of barley varieties to establishment of subcuticular mycelia by *Rhynchosporium secalis* . Transactions of the British Mycological Society, 57, 233–240.

[pce13350-bib-0011] Bachmann, A. , Hause, B. , Maucher, H. , Garbe, E. , VörÖs, K. , Weichert, H. , … Feussner, I. (2002). Jasmonate‐induced lipid peroxidation in barley leaves initiated by distinct 13‐LOX forms of chloroplasts. Biological Chemistry, 383, 1645–1657. 12452441 10.1515/BC.2002.185

[pce13350-bib-0012] Berens, M. L. , Berry, H. M. , Mine, A. , Argueso, C. T. , & Tsuda, K. (2017). Evolution of hormone signaling networks in plant defense. Annual Review of Phytopathology, 55, 401–425. 10.1146/annurev-phyto-080516-035544 28645231

[pce13350-bib-0013] Beyhl, D. , Hörtensteiner, S. , Martinoia, E. , Farmer, E. E. , Fromm, J. , Marten, I. , & Hedrich, R. (2009). The *fou2* mutation in the major vacuolar cation channel TPC1 confers tolerance to inhibitory luminal calcium. The Plant Journal, 58, 715–723. 19298454 10.1111/j.1365-313X.2009.03820.x

[pce13350-bib-0014] Bloem, E. , Haneklaus, S. , Salac, I. , Wickenhäuser, P. , & Schnug, E. (2007). Facts and fiction about sulfur metabolism in relation to plant–pathogen interactions. Plant Biology, 9, 596–607. 17853360 10.1055/s-2007-965420

[pce13350-bib-0015] Bonaventure, G. , Gfeller, A. , Proebsting, W. M. , Hörtensteiner, S. , Chételat, A. , Martinoia, E. , & Farmer, E. E. (2007). A gain‐of‐function allele of TPC1 activates oxylipin biogenesis after leaf wounding in *Arabidopsis* . Plant Journal, 49, 889–898. 17253984 10.1111/j.1365-313X.2006.03002.x

[pce13350-bib-0016] Cao, S. , Su, L. , & Fang, Y. (2006). Evidence for involvement of jasmonic acid in the induction of leaf senescence by potassium deficiency in *Arabidopsis* . Canadian Journal of Botany, 333, 328–333.

[pce13350-bib-0017] Carden, D. E. , Walker, D. J. , Flowers, T. J. , & Miller, A. J. (2003). Single‐cell measurements of the contributions of cytosolic Na^+^ and K^+^ to salt tolerance. Plant Physiology, 131, 676–683. 12586891 10.1104/pp.011445 PMC166843

[pce13350-bib-0018] Chaudhry, B. , Müller‐Uri, F. , Mills, V. , Gough, S. , Simpson, D. , Skriver, K. , & Mundy, J. (1994). The barley 60 kDa jasmonate induced protein (JIP60) is a novel ribosome inactivating protein. The Plant Journal, 6, 815–824. 7849755 10.1046/j.1365-313x.1994.6060815.x

[pce13350-bib-0019] Chérel, I. , Lefoulon, C. , Boeglin, M. , & Sentenac, H. (2014). Molecular mechanisms involved in plant adaptation to low K^+^ availability. Journal of Experimental Botany, 65, 833–848. 24293613 10.1093/jxb/ert402

[pce13350-bib-0020] Cowley, T. , & Walters, D. (2005). Local and systemic effects of oxylipins on powdery mildew infection in barley. Pest Management Science, 61, 572–576. 15668923 10.1002/ps.1026

[pce13350-bib-0021] Dar, T. A. , Uddin, M. , Khan, M. M. A. , Hakeem, K. R. , & Jaleel, H. (2015). Jasmonates counter plant stress: A review. Environmental and Experimental Botany, 115, 49–57.

[pce13350-bib-0022] Datnoff, L. E. , & Elmer, W. H. (2007). Mineral nutrition and plant disease. St. Paul, Minnesota: American Phytopathological Society (APS Press).

[pce13350-bib-0023] Dave, A. , Hernández, M. L. , He, Z. , Andriotis, V. M. E. , Vaistij, F. E. , Larson, T. R. , & Graham, I. A. (2011). 12‐Oxo‐phytodienoic acid accumulation during seed development represses seed germination in arabidopsis. The Plant Cell, 23, 583–599. 21335376 10.1105/tpc.110.081489 PMC3077774

[pce13350-bib-0024] Delker, C. , Stenzel, I. , Hause, B. , Miersch, O. , Feussner, I. , & Wasternack, C. (2006). Jasmonate biosynthesis in *Arabidopsis thaliana*—Enzymes, products, regulation. Plant Biology, 8, 297–306. 16807821 10.1055/s-2006-923935

[pce13350-bib-0025] Devoto, A. , & Turner, J. G. (2005). Jasmonate‐regulated arabidopsis stress signalling network. Physiologia Plantarum, 123, 161–172.

[pce13350-bib-0026] Ding, L.‐N. , Yang, G.‐X. , Yang, R.‐Y. , Cao, J. , & Zhou, Y. (2016). Investigating interactions of salicylic acid and jasmonic acid signaling pathways in monocots wheat. Physiological and Molecular Plant Pathology, 93, 67–74.

[pce13350-bib-0027] Dunaeva, M. , Goebel, C. , Wasternack, C. , Parthier, B. , & Goerschen, E. (1999). The jasmonate‐induced 60 kDa protein of barley exhibits *N* ‐glycosidase activity in vivo. FEBS Letters, 452, 263–266. 10386603 10.1016/s0014-5793(99)00645-6

[pce13350-bib-0028] Ellis, C. , Karafyllidis, I. , & Turner, J. G. (2002). Constitutive activation of jasmonate signaling in an arabidopsis mutant correlates with enhanced resistance to *Erysiphe cichoracearum*, *Pseudomonas syringae*, and *Myzus persicae* . Molecular Plant‐Microbe Interactions, 15, 1025–1030. 12437300 10.1094/MPMI.2002.15.10.1025

[pce13350-bib-0029] Ellis, C. , & Turner, J. G. (2001). The arabidopsis mutant *cev1* has constitutively active jasmonate and ethylene signal pathways and enhanced resistance to pathogens. The Plant Cell, 13, 1025–1033. 11340179 10.1105/tpc.13.5.1025 PMC135553

[pce13350-bib-0030] Evans, N. H. , Gottlieb, H. , & Bach, D. (2003). Modulation of guard cell plasma membrane potassium currents by methyl jasmonate. Plant Physiology, 131, 8–11. 12529509 10.1104/pp.014266 PMC1540276

[pce13350-bib-0031] Fricke, W. , Hinde, P. , Leigh, R. , & Tomos, A. D. (1995). Vacuolar solutes in the upper epidermis of barley leaves. Planta, 196, 40–49.

[pce13350-bib-0032] Fricke, W. , Leigh, R. , & Deri Tomos, A. (1994a). Concentrations of inorganic and organic solutes in extracts from individual epidermal, mesophyll and bundle‐sheath cells of barley leaves. Planta, 192, 310–316.

[pce13350-bib-0033] Fricke, W. , Leigh, R. , & Deri Tomos, A. (1994b). Epidermal solute concentrations and osmolality in barley leaves studied at the single‐cell level. Planta, 192, 317–323.

[pce13350-bib-0034] Glawe, D. A. (2008). The powdery mildews: A review of the world's most familiar (yet poorly known) plant pathogens. Annual Review of Phytopathology, 46, 27–51. 10.1146/annurev.phyto.46.081407.104740 18680422

[pce13350-bib-0035] Glazebrook, J. (2005). Contrasting mechanisms of defense against biotrophic and necrotrophic pathogens. Annual Review of Phytopathology, 43, 205–227. 10.1146/annurev.phyto.43.040204.135923 16078883

[pce13350-bib-0036] Gupta, N. , Debnath, S. , Sharma, S. , Sharma, P. , & Purohit, J. (2017). Role of nutrients in controlling the plant diseases in sustainable agriculture In Agriculturally important microbes for sustainable agriculture (pp. 217–262). Singapore: Springer Singapore.

[pce13350-bib-0037] Halkier, B. A. , & Gershenzon, J. (2006). Biology and biochemistry of glucosinolates. Annual Review of Plant Biology, 57, 303–333. 10.1146/annurev.arplant.57.032905.105228 16669764

[pce13350-bib-0038] Huber, D. , Römheld, V. , & Weinmann, M. (2012). Chapter 10—Relationship between nutrition, plant diseases and pests In Marschner P. (Ed.), Marschner's mineral nutrition of higher plants (3rd ed.) (pp. 283–298). London U.K.: Academic Press.

[pce13350-bib-0039] Imas, P. , & Magen, H. (2000). Potash facts in brief—Potassium. An essential nutrient. Bern, Switzerland and Haryana, India: International Potash Institute.

[pce13350-bib-0040] Karley, A. J. , Leigh, R. A. , & Sanders, D. (2000). Where do all the ions go? The cellular basis of differential ion accumulation in leaf cells. Trends in Plant Science, 5, 465–470. 11077254 10.1016/s1360-1385(00)01758-1

[pce13350-bib-0041] Karley, A. J. , & White, P. J. (2009). Moving cationic minerals to edible tissues: Potassium, magnesium, calcium. Current Opinion in Plant Biology, 12, 291–298. 19481494 10.1016/j.pbi.2009.04.013

[pce13350-bib-0042] Kazan, K. , & Lyons, R. (2014). Intervention of phytohormone pathways by pathogen effectors. The Plant Cell, 26, 2285–2309. 24920334 10.1105/tpc.114.125419 PMC4114936

[pce13350-bib-0043] Kazan, K. , & Manners, J. M. (2008). Jasmonate signaling: Toward an integrated view. Plant Physiology, 146, 1459–1468. 18390489 10.1104/pp.107.115717 PMC2287326

[pce13350-bib-0044] Leigh, R. A. , Chater, M. , Storey, R. , & Johnston, A. P. (1986). Accumulation and subcellular distribution of cations in relation to the growth of potassium‐deficient barley. Plant, Cell and Environment, 9, 595–604.

[pce13350-bib-0045] Loake, G. , & Grant, M. (2007). Salicylic acid in plant defence—The players and protagonists. Current Opinion in Plant Biology, 10, 466–472. 17904410 10.1016/j.pbi.2007.08.008

[pce13350-bib-0046] Lyons, R. , Manners, J. M. , & Kazan, K. (2013). Jasmonate biosynthesis and signaling in monocots: A comparative overview. Plant Cell Reports, 32, 815–827. 23455708 10.1007/s00299-013-1400-y

[pce13350-bib-0047] Maffei, M. E. , Mithöfer, A. , & Boland, W. (2007). Before gene expression: Early events in plant–insect interaction. Trends in Plant Science, 12, 310–316. 17596996 10.1016/j.tplants.2007.06.001

[pce13350-bib-0048] Moscou, M. J. , Lauter, N. , Steffenson, B. , Wise, R. P. , & Soller, M. (2011). Quantitative and qualitative stem rust resistance factors in barley are associated with transcriptional suppression of defense regulons. PLoS Genetics, 7, e1002208. 21829384 10.1371/journal.pgen.1002208 PMC3145622

[pce13350-bib-0049] Mur, L. A. J. , Kenton, P. , Atzorn, R. , Miersch, O. , & Wasternack, C. (2006). The outcomes of concentration‐specific interactions between salicylate and jasmonate signaling include synergy, antagonism, and oxidative stress leading to cell death. Plant Physiology, 140. 10.1104/pp.105.072348 PMC1326048 16377744

[pce13350-bib-0050] Newton, A. C. (1989). Genetic adaptation of *Erysiphc graminis* f. sp. *Hordei* to barley with partial resistance. Journal of Phytopathology, 126, 133–148.

[pce13350-bib-0051] Newton, A. C. , Fitt, B. D. L. , Atkins, S. D. , Walters, D. R. , & Daniell, T. J. (2010). Pathogenesis, parasitism and mutualism in the trophic space of microbe–plant interactions. Trends in Microbiology, 18, 365–373. 20598545 10.1016/j.tim.2010.06.002

[pce13350-bib-0052] Newton, A. C. , Hackett, C. A. , & Guy, D. C. (1998). Diversity and complexity of *Erysiphe graminis* f. sp. *hordei* collected from barley cultivar mixtures or barley plots treated with a resistance elicitor. European Journal of Plant Pathology, 104, 925–931.

[pce13350-bib-1052] Newton, A. C. , Searle, J. , Guy, D. C. , Hackett, C. A. , & Cooke, D. E. L. (2001). Variability in pathotype, aggressiveness, RAPD profile, and rDNA ITS1 sequences of UK isolates of Rhynchosporium secalis. Journal of Plant Diseases and Protection, 108, 446–458.

[pce13350-bib-0053] Pathak, R. K. , Baunthiyal, M. , Pandey, N. , Pandey, D. , & Kumar, A. (2017). Modeling of the jasmonate signaling pathway in *Arabidopsis thaliana* with respect to pathophysiology of Alternaria blight in Brassica. Scientific Reports, 7, 16790. 29196636 10.1038/s41598-017-16884-3 PMC5711873

[pce13350-bib-0054] Peiter, E. , Maathuis, F. J. M. , Mills, L. N. , Knight, H. , Pelloux, J. , Hetherington, A. M. , & Sanders, D. (2005). The vacuolar Ca^2+^‐activated channel TPC1 regulates germination and stomatal movement. Nature, 434, 404–408. 15772667 10.1038/nature03381

[pce13350-bib-0055] Per, T. S. , Khan, M. I. R. , Anjum, N. A. , Masood, A. , Hussain, S. J. , & Khan, N. A. (2018). Jasmonates in plants under abiotic stresses: Crosstalk with other phytohormones matters. Environmental and Experimental Botany, 145, 104–120.

[pce13350-bib-0056] Perrenoud, S. (1990). Potassium and plantation health (Vol. 3). Basel, Switzerland: International Potash Institute.

[pce13350-bib-0057] Piffanelli, P. , Ramsay, L. , Waugh, R. , Benabdelmouna, A. , D'Hont, A. , Hollricher, K. , … Panstruga, R. (2004). A barley cultivation‐associated polymorphism conveys resistance to powdery mildew. Nature, 430, 887–891. 15318221 10.1038/nature02781

[pce13350-bib-0058] Pimentel, D. (2005). Environmental and economic costs of the application of pesticides primarily in the United States. Environment, Development and Sustainability, 7, 229–252.

[pce13350-bib-0059] Prabhu, A. S. , Fageria, N. K. , Huber, D. M. , & Rodrigues, F. A. (2007). Potassium nutrition and plant diseases In Datnoff L. E., Elmer W. H., & Huber D. M. (Eds.), Mineral nutrition and plant disease (pp. 57–78). Saint Paul, USA: The American Phytopathological Society Press.

[pce13350-bib-0060] Ranf, S. , Wünnenberg, P. , Lee, J. , Becker, D. , Dunkel, M. , Hedrich, R. , … Dietrich, P. (2007). Loss of the vacuolar cation channel, AtTPC1, does not impair Ca^2+^ signals induced by abiotic and biotic stresses. The Plant Journal, 53, 287–299. 18028262 10.1111/j.1365-313X.2007.03342.x

[pce13350-bib-0061] Reinbothe, S. , Reinbothe, C. , Lehmann, J. , Becker, W. , Apel, K. , & Parthier, B. (1994). JIP60, a methyl jasmonate‐induced ribosome‐inactivating protein involved in plant stress reactions. Proceedings of the National Academy of Sciences of the United States of America, 91, 7012–7016. 8041737 10.1073/pnas.91.15.7012 PMC44328

[pce13350-bib-0062] Savary, S. , Ficke, A. , Aubertot, J.‐N. , & Hollier, C. (2012). Crop losses due to diseases and their implications for global food production losses and food security. Food Security, 4, 519–537.

[pce13350-bib-0063] Schweizer, P. , Gees, R. , & Mosinger, E. (1993). Effect of jasmonic acid on the interaction of barley (*Hordeum vulgare* L.) with the powdery mildew *Erysiphe graminis* f. sp. *hordei* . Plant Physiology, 102, 503–511. 12231839 10.1104/pp.102.2.503 PMC158805

[pce13350-bib-0064] Seeholzer, S. , Tsuchimatsu, T. , Jordan, T. , Bieri, S. , Pajonk, S. , Yang, W. , … Schulze‐Lefert, P. (2010). Diversity at the Mla powdery mildew resistance locus from cultivated barley reveals sites of positive selection. Molecular Plant–Microbe Interactions, 23, 497–509. 20192836 10.1094/MPMI-23-4-0497

[pce13350-bib-0065] Shin, R. , & Schachtman, D. P. (2004). Hydrogen peroxide mediates plant root cell response to nutrient deprivation. Proceedings of the National Academy of Sciences, 101, 8827–8832. 10.1073/pnas.0401707101 PMC423280 15173595

[pce13350-bib-0066] Shyu, C. , & Brutnell, T. P. (2015). Growth‐defence balance in grass biomass production: The role of jasmonates. Journal of Experimental Botany, 66, 4165–4176. 25711704 10.1093/jxb/erv011

[pce13350-bib-0067] Steiner‐Lange, S. , Fischer, A. , Boettcher, A. , Rouhara, I. , Liedgens, H. , Schmelzer, E. , & Knogge, W. (2003). Differential defense reactions in leaf tissues of barley in response to infection by *Rhynchosporium secalis* and to treatment with a fungal avirulence gene product. Molecular Plant‐Microbe Interactions: MPMI, 16, 893–902. 14558691 10.1094/MPMI.2003.16.10.893

[pce13350-bib-0068] Sulpice, R. , Pyl, E.‐T. , Ishihara, H. , Trenkamp, S. , Steinfath, M. , Witucka‐Wall, H. , … Stitt, M. (2009). Starch as a major integrator in the regulation of plant growth. Proceedings of the National Academy of Sciences of the United States of America, 106, 10348–10353. 19506259 10.1073/pnas.0903478106 PMC2693182

[pce13350-bib-0069] Sulpice, R. , Trenkamp, S. , Steinfath, M. , Usadel, B. , Gibon, Y. , Witucka‐Wall, H. , … Stitt, M. (2010). Network analysis of enzyme activities and metabolite levels and their relationship to biomass in a large panel of *Arabidopsis* accessions. The Plant Cell, 22, 2872–2893. 20699391 10.1105/tpc.110.076653 PMC2947169

[pce13350-bib-0070] Tegtmeier, E. M. , & Duffy, M. D. (2004). External costs of agricultural production in the United States. International Journal of Agricultural Sustainability, 2, 1–20.

[pce13350-bib-0071] Thaler, J. S. , Humphrey, P. T. , & Whiteman, N. K. (2012). Evolution of jasmonate and salicylate signal crosstalk. Trends in Plant Science, 17, 260–270. 22498450 10.1016/j.tplants.2012.02.010

[pce13350-bib-0072] Thaler, J. S. , Owen, B. , & Higgins, V. J. (2004). The role of the jasmonate response in plant susceptibility to diverse pathogens with a range of lifestyles. Plant Physiology, 135, 530–538. 15133157 10.1104/pp.104.041566 PMC429405

[pce13350-bib-0073] Thordal‐Christensen, H. , Zhang, Z. , Wei, Y. , & Collinge, D. B. (1997). Subcellular localization of H_2_O_2_ in plants. H_2_O_2_ accumulation in papillae and hypersensitive response during the barley–powdery mildew interaction. The Plant Journal, 11, 1187–1194.

[pce13350-bib-0074] Troufflard, S. , Mullen, W. , Larson, T. R. , Graham, I. A. , Crozier, A. , Amtmann, A. , & Armengaud, P. (2010). Potassium deficiency induces the biosynthesis of oxylipins and glucosinolates in *Arabidopsis thaliana* . BMC Plant Biology, 10, 172. 20701801 10.1186/1471-2229-10-172 PMC3017790

[pce13350-bib-0075] Truman, W. , Bennett, M. H. , Kubigsteltig, I. , Turnbull, C. , & Grant, M. (2007). Arabidopsis systemic immunity uses conserved defense signaling pathways and is mediated by jasmonates. Proceedings of the National Academy of Sciences of the United States of America, 104, 1075–1080. 17215350 10.1073/pnas.0605423104 PMC1783366

[pce13350-bib-0076] Tschoep, H. , Gibon, Y. , Carillo, P. , Armengaud, P. , Szecowka, M. , Nunes‐Nesi, A. , … Stitt, M. (2009). Adjustment of growth and central metabolism to a mild but sustained nitrogen‐limitation in *Arabidopsis* . Plant, Cell and Environment, 32, 300–318. 10.1111/j.1365-3040.2008.01921.x 19054347

[pce13350-bib-0077] Volkov, V. , Boscari, A. , Clément, M. , Miller, A. J. , Amtmann, A. , & Fricke, W. (2009). Electrophysiological characterization of pathways for K^+^ uptake into growing and non‐growing leaf cells of barley. Plant, Cell and Environment, 32, 1778–1790. 10.1111/j.1365-3040.2009.02034.x 19682290

[pce13350-bib-0078] Wakeel, A. , Gul, M. , & Zörb, C. (2016). Potassium for sustainable agriculture In Soil science: Agricultural and environmental prospectives (pp. 159–182). Cham: Springer International Publishing.

[pce13350-bib-0079] Walker, D. J. , Leigh, R. A. , & Miller, A. J. (1996). Potassium homeostasis in vacuolate plant cells. Proceedings of the National Academy of Sciences of the United State of America, 93, 10510–10514. 10.1073/pnas.93.19.10510 PMC38416 11607707

[pce13350-bib-0080] Walters, D. , Cowley, T. , & Mitchell, A. (2002). Methyl jasmonate alters polyamine metabolism and induces systemic protection against powdery mildew infection in barley seedlings. Journal of Experimental Botany, 53, 747–756. 11886895 10.1093/jexbot/53.369.747

[pce13350-bib-0081] Walters, D. R. , Havis, N. D. , Paterson, L. , Taylor, J. , Walsh, D. J. , & Sablou, C. (2014). Control of foliar pathogens of spring barley using a combination of resistance elicitors. Frontiers in Plant Science, 5, 241. 24904629 10.3389/fpls.2014.00241 PMC4036063

[pce13350-bib-0082] Walters, D. R. , Paterson, L. , Sablou, C. , & Walsh, D. J. (2011). Existing infection with *Rhynchosporium secalis* compromises the ability of barley to express induced resistance. European Journal of Plant Pathology, 130, 73–82.

[pce13350-bib-0083] Wang, Y. , & Wu, W.‐H. (2013). Potassium transport and signaling in higher plants. Annual Review of Plant Biology, 64, 451–476. 10.1146/annurev-arplant-050312-120153 23330792

[pce13350-bib-0084] Wasternack, C. , & Hause, B. (2013). Jasmonates: Biosynthesis, perception, signal transduction and action in plant stress response, growth and development. An update to the 2007 review in *Annals of Botany* . Annals of Botany, 111, 1021–1058. 23558912 10.1093/aob/mct067 PMC3662512

[pce13350-bib-0085] Wasternack, C. , Parthier, B. , & Mullet, J. E. (1997). Jasmonate‐signalled plant gene expression. Trends in Plant Science, 2, 302–307.

[pce13350-bib-0086] Wasternack, C. , & Strnad, M. (2016). Jasmonate signaling in plant stress responses and development—Active and inactive compounds. New Biotechnology, 33, 604–613. 26581489 10.1016/j.nbt.2015.11.001

[pce13350-bib-0087] Weidhase, R. A. , Kramell, H. M. , Lehmann, J. , Liebisch, H. W. , Lerbs, W. , & Parthier, B. (1987). Methyljasmonate‐induced changes in the polypeptide pattern of senescing barley leaf segments. Plant Science, 51, 177–186.

[pce13350-bib-0088] Weiskorn, C. , Kramer, M. , Ordon, F. , & Friedt, W. (2002). Induced resistance in barley (*Hordeum vulgare* L.) against *Rhynchosporium secalis* and Barley Yellow Dwarf Virus (BYDV). IOBC/wprs Bull., 25, 149–153.

[pce13350-bib-0089] White, P. J. , Broadley, M. R. , & Gregory, P. J. (2012). Managing the nutrition of plants and people. Applied and Environmental Soil Science, 2012, 1–13.

[pce13350-bib-0090] White, P. J. , & Karley, A. J. (2010). Potassium In Hell R. & Mendel R. R. (Eds.), Cell biology of metals and nutrients (pp. 199–224). Berlin, Heidelberg: Springer.

[pce13350-bib-0091] White, P. J. , & Veneklaas, E. J. (2012). Nature and nurture: The importance of seed phosphorus content. Plant and Soil, 357, 1–8.

[pce13350-bib-0092] Wise, R. P. , Lauter, N. , Szabo, L. , & Schweizer, P. (2009). Genomics of biotic interactions in the Triticeae In Genetics and genomics of the Triticeae (pp. 559–609). New York, NY: Springer US.

[pce13350-bib-0093] Yan, J. , Li, S. , Gu, M. , Yao, R. , Li, Y. , Chen, J. , … Xie, D. (2016). Endogenous bioactive jasmonate is composed of a set of (+)‐7‐iso‐JA–amino acid conjugates. Plant Physiology, 172, 2154–2164. 27756820 10.1104/pp.16.00906 PMC5129707

[pce13350-bib-0094] Yan, J. B. , Zhang, C. , Gu, M. , Bai, Z. Y. , Zhang, W. G. , Qi, T. C. , … Xie, D. (2009). The *Arabidopsis* CORONATINE INSENSITIVE1 protein is a jasmonate receptor. Plant Cell, 21, 2220–2236. 19717617 10.1105/tpc.109.065730 PMC2751961

[pce13350-bib-0095] Yan, S. , McLamore, E. S. , Dong, S. , Gao, H. , Taguchi, M. , Wang, N. , … Shen, Y. (2015). The role of plasma membrane H^+^‐ATPase in jasmonate‐induced ion fluxes and stomatal closure in *Arabidopsis thaliana* . The Plant Journal, 83, 638–649. 26088926 10.1111/tpj.12915

[pce13350-bib-0096] Yang, Y. , Shah, J. , & Klessig, D. F. (1997). Signal perception and transduction in plant defense responses. Genes & Development, 11, 1621–1639. 9224713 10.1101/gad.11.13.1621

[pce13350-bib-0097] Zellerhoff, N. , Himmelbach, A. , Dong, W. , Bieri, S. , Schaffrath, U. , & Schweizer, P. (2010). Nonhost resistance of barley to different fungal pathogens is associated with largely distinct, quantitative transcriptional responses. Plant Physiology, 152, 2053–2066. 20172964 10.1104/pp.109.151829 PMC2850024

