# Supplementary material for: Contrasting nutrient–disease relationships: Potassium gradients in barley leaves have opposite effects on two fungal pathogens with different sensitivities to jasmonic acid
Source: Plant Cell Environ. 2018 Jun 29;41(10):2357–72. doi: 10.1111/pce.13350 (PMC6175101; doi:10.1111/pce.13350)
Supplement: Supplementary file 1 — Figure S1. Barley leaves and leaf zones. A: Numbering of leaves. B: Leaf segments of second leaf harvested for the analysis Figure S2. Pathogen symptoms and inoculation procedure. A: Lesions caused by R. commune 12 days after inoculation. B: Colonies of B. graminis 12 days after inoculation. C: Setup for applying B. graminis spores to leaf segments. D: Appearance of un‐inoculated leaf segments after15 days on the inoculation plates. Figure 3S. Growth and K content of barley roots in control and low‐K conditions. Length (A), fresh weight (B) and K concentration (C) of roots from barley plants grown in control (black symbols) or ‐K (open symbols) media. Five plants were harvested for each time point, the means (± SE) from three independently grown and treated plant batches are shown (n=3). Pictures of representative roots and shoots 20 days after K deprivation are shown in D, E, and F. Figure S4: Dendrogram showing the relationship between LOX2 gene sequences. Locus identifiers are shown in brackets and bootstrap values in italics. Figure S5. Identification of suitable constitutive reference gene. A: Variation across samples of transcript levels determined from Ct values in qPCR for UBQ, GAPDH and α‐TUB. B, C: Frequency distribution of transcript levels obtained in qPCR for GAPDH (B) and α‐TUB (C). RNA was prepared from barley plants grown in full nutrient control and low‐K nutrient solution for 3, 6, 9, 12 and 15 days, and transcript levels were normalised to day‐3 control. Figure S6: Oxylipin concentrations of leaf segments Oxylipin concentrations in leaf segments from barley plants grown in control (black bars) or ‐K (open bars) media. Leaf tissue of tip (A), middle (B) and base (C) segments from the emerged blade of the second leaf of 20 plants was pooled for each sample, and means (± SE) of three independently grown and treated batches of plants are shown. For abbreviations of oxylipin names, see text. ‘Oxy’ is the sum of all measured oxylipins. Figure S7 Current [file PCE-41-2357-s001.pptx]

## Slide 1
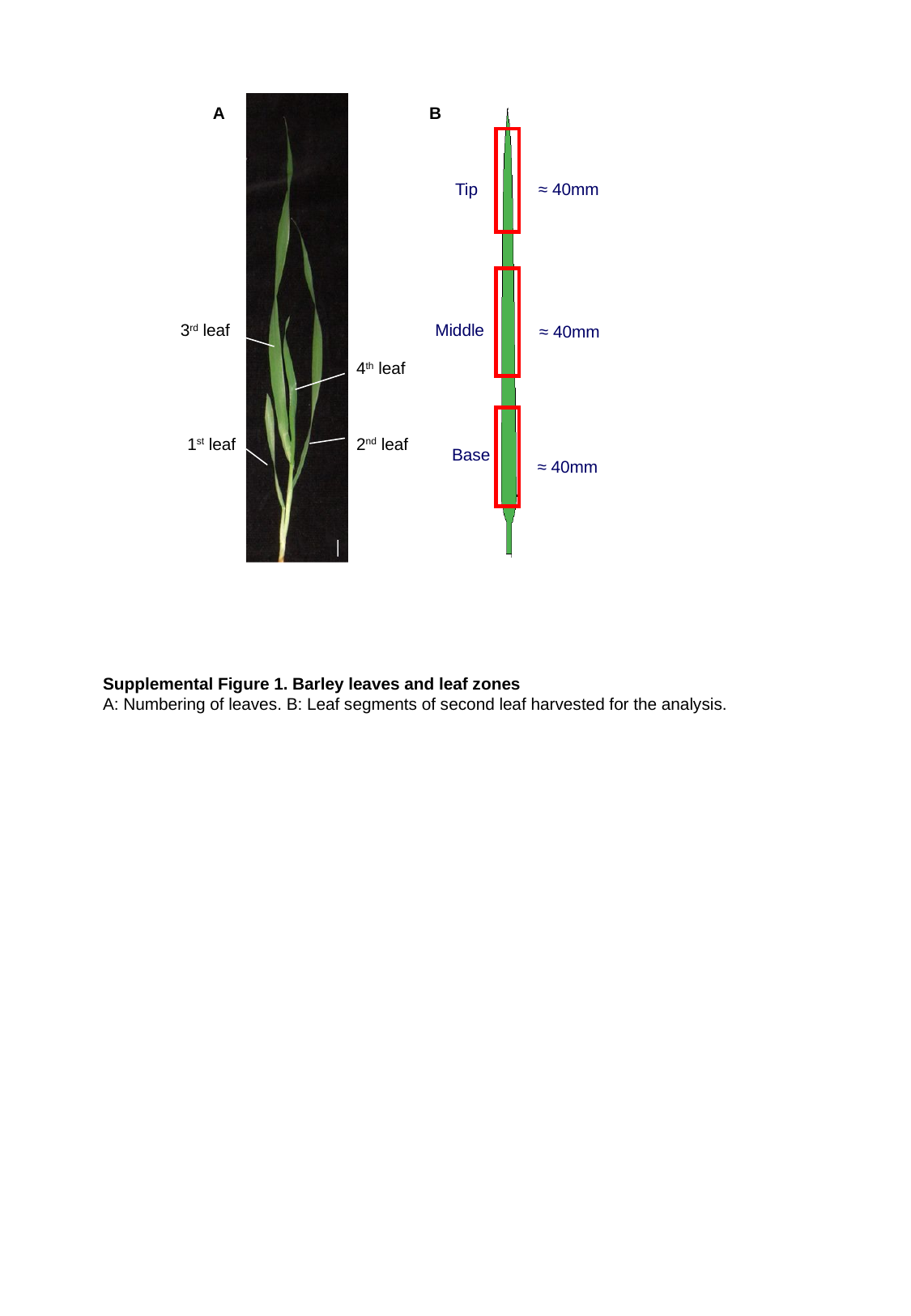

A
B
Tip
≈ 40mm
3rd leaf
Middle
≈ 40mm
4th leaf
1st leaf
2nd leaf
Base
≈ 40mm
Supplemental Figure 1. Barley leaves and leaf zones
A: Numbering of leaves. B: Leaf segments of second leaf harvested for the analysis.

## Slide 2
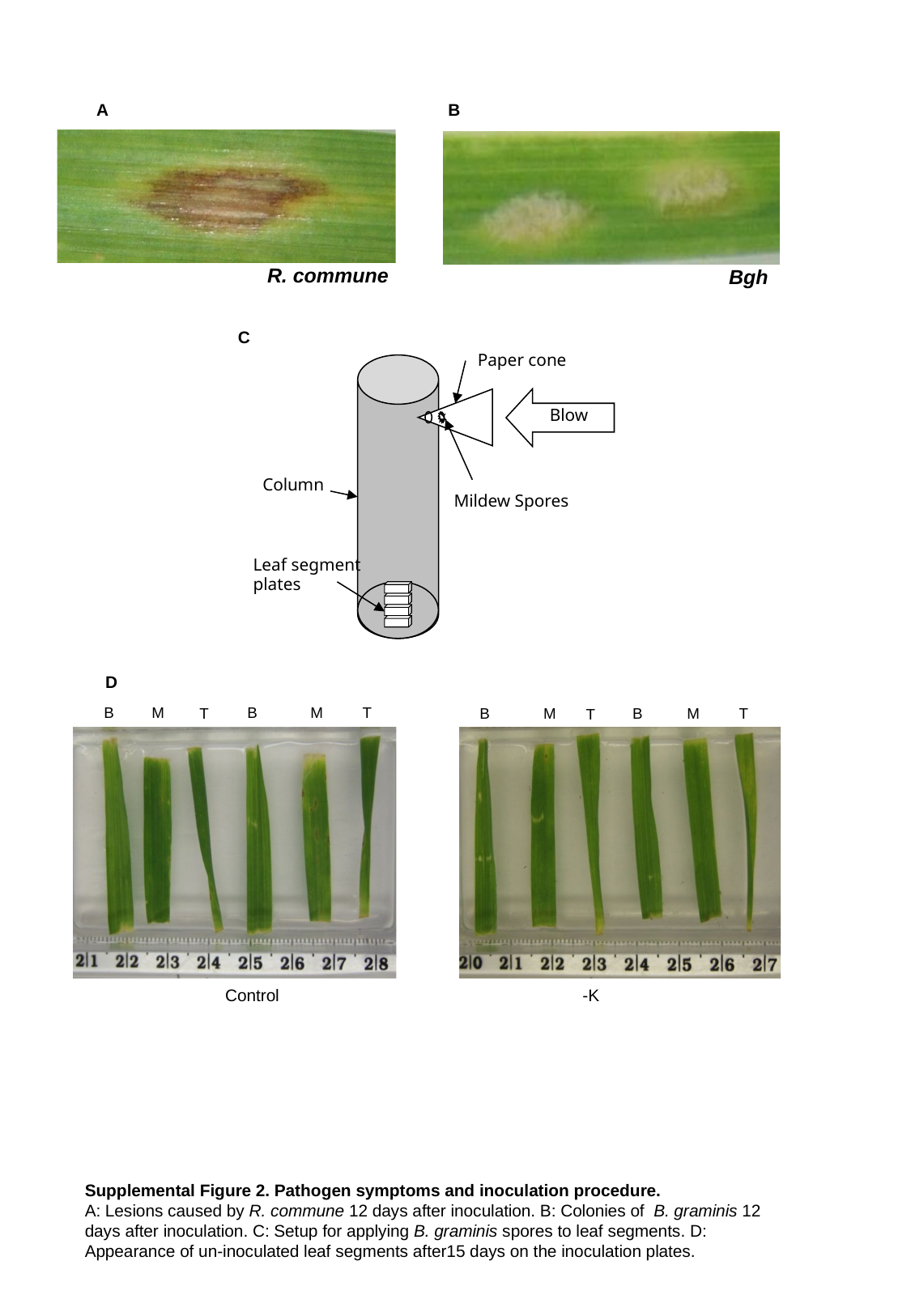

A
B
R. commune
Bgh
C
Paper cone
Blow
Column
Mildew Spores
Leaf segment plates
D
B
M
B
M
T
T
B
M
B
M
T
T
Control
-K
Supplemental Figure 2. Pathogen symptoms and inoculation procedure.
A: Lesions caused by R. commune 12 days after inoculation. B: Colonies of B. graminis 12 days after inoculation. C: Setup for applying B. graminis spores to leaf segments. D: Appearance of un-inoculated leaf segments after15 days on the inoculation plates.

## Slide 3
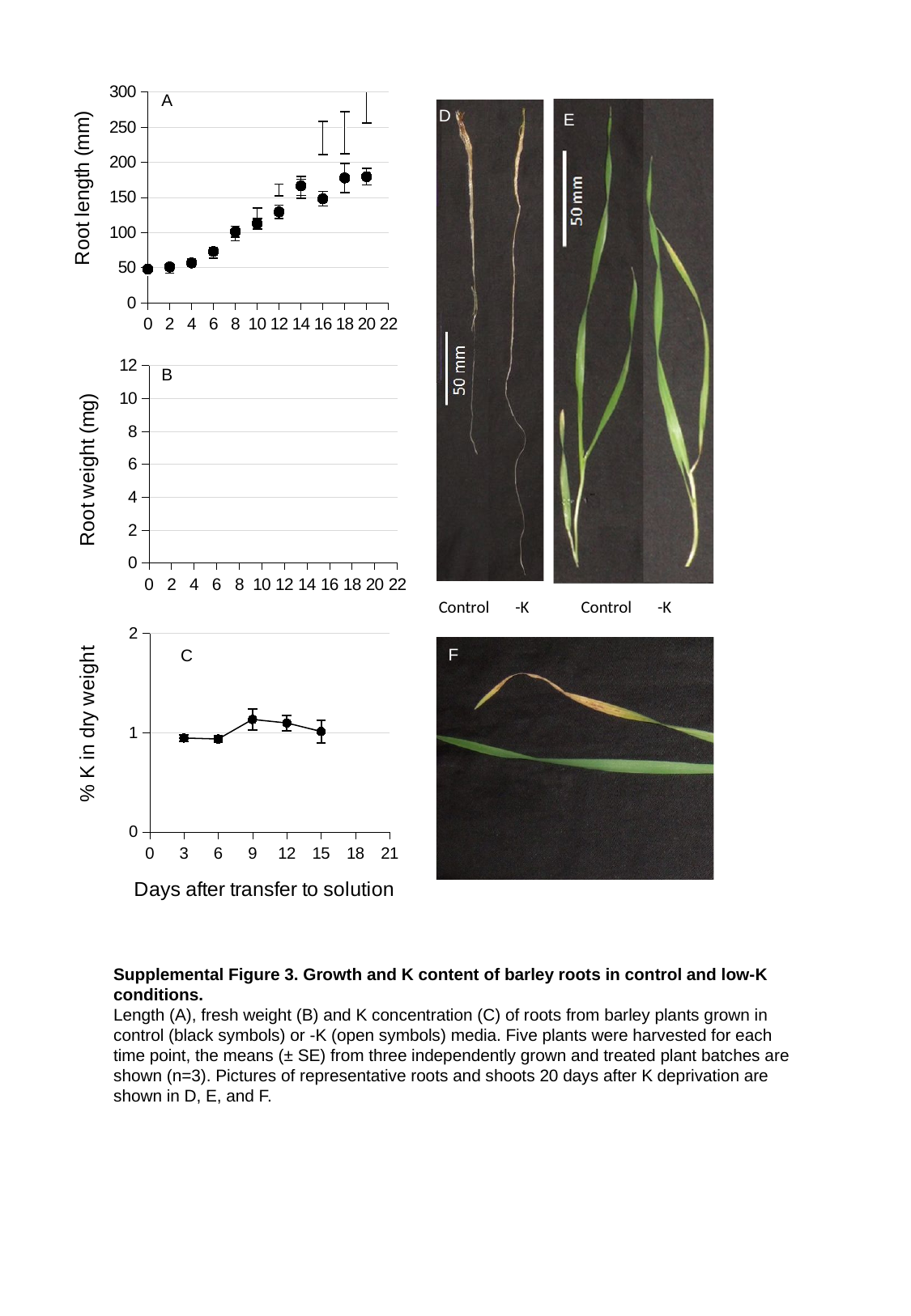

### Chart
| Category | | |
|---|---|---|A
D
E
### Chart
| Category | | |
|---|---|---|B
Control
-K
Control
-K
### Chart
| Category | | |
|---|---|---|
F
C
Supplemental Figure 3. Growth and K content of barley roots in control and low-K conditions.
Length (A), fresh weight (B) and K concentration (C) of roots from barley plants grown in control (black symbols) or -K (open symbols) media. Five plants were harvested for each time point, the means (± SE) from three independently grown and treated plant batches are shown (n=3). Pictures of representative roots and shoots 20 days after K deprivation are shown in D, E, and F.

## Slide 4
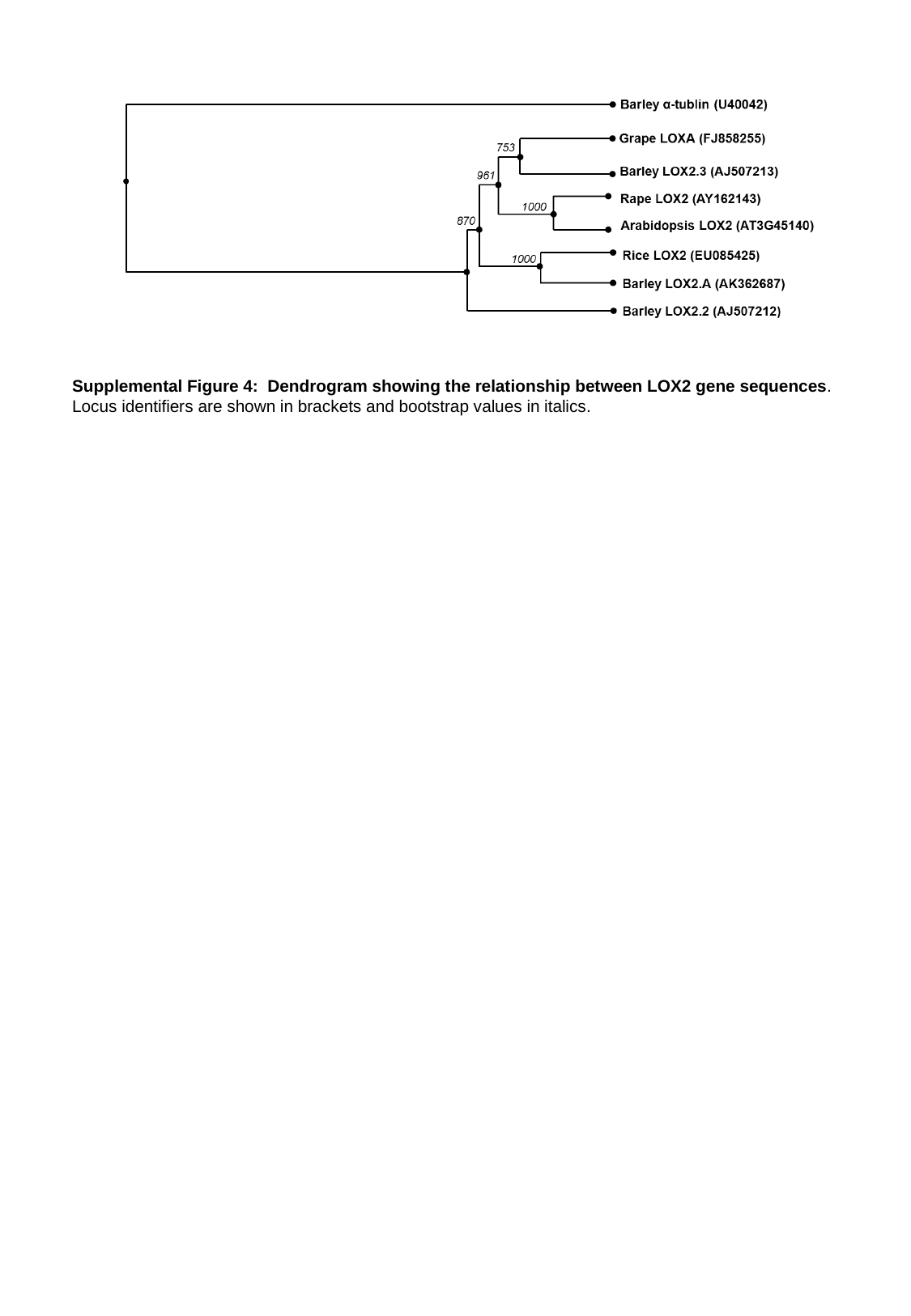

Supplemental Figure 4: Dendrogram showing the relationship between LOX2 gene sequences. Locus identifiers are shown in brackets and bootstrap values in italics.

## Slide 5
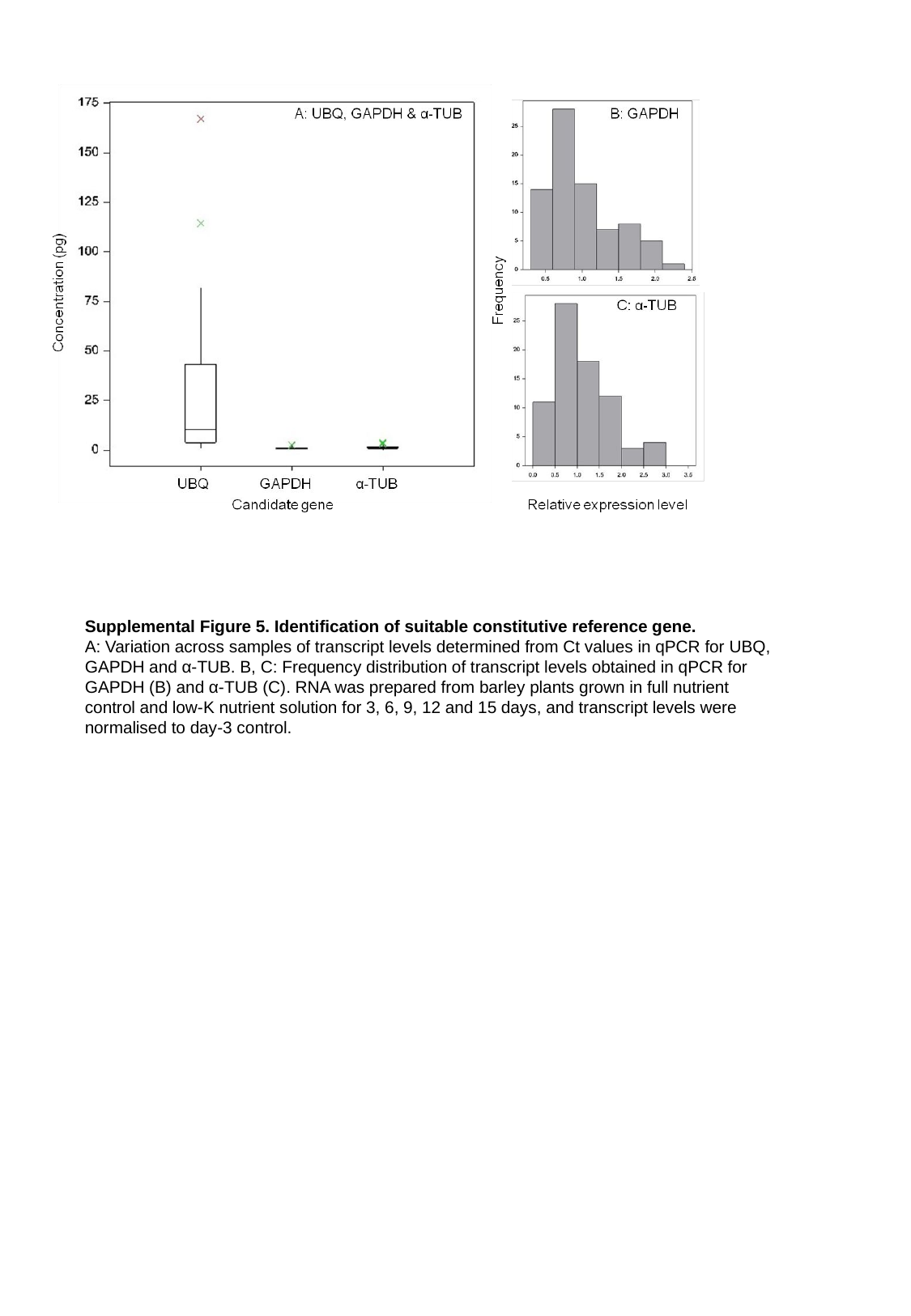

Supplemental Figure 5. Identification of suitable constitutive reference gene.
A: Variation across samples of transcript levels determined from Ct values in qPCR for UBQ, GAPDH and α-TUB. B, C: Frequency distribution of transcript levels obtained in qPCR for GAPDH (B) and α-TUB (C). RNA was prepared from barley plants grown in full nutrient control and low-K nutrient solution for 3, 6, 9, 12 and 15 days, and transcript levels were normalised to day-3 control.

## Slide 6
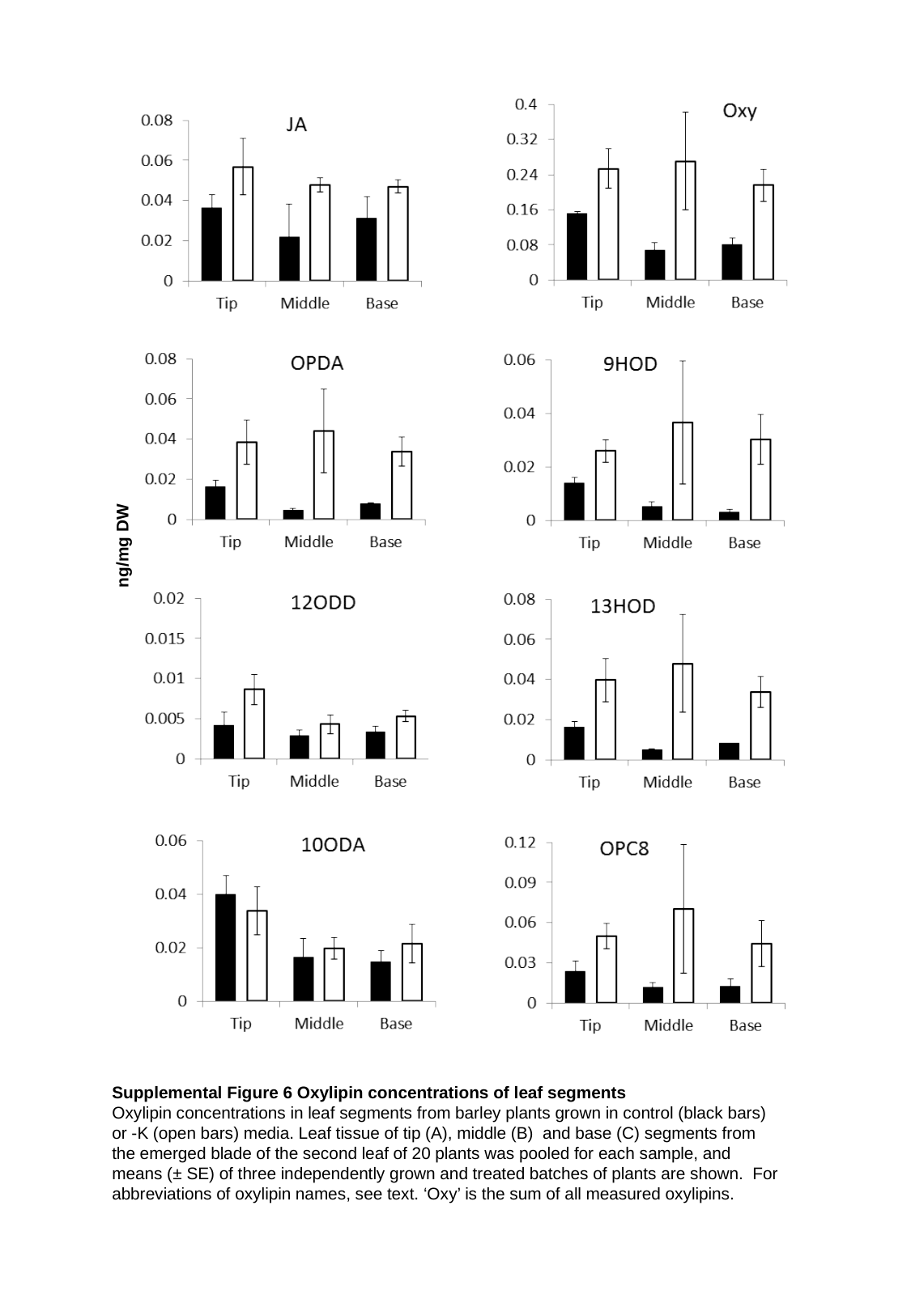

ng/mg DW
Supplemental Figure 6 Oxylipin concentrations of leaf segments
Oxylipin concentrations in leaf segments from barley plants grown in control (black bars) or -K (open bars) media. Leaf tissue of tip (A), middle (B) and base (C) segments from the emerged blade of the second leaf of 20 plants was pooled for each sample, and means (± SE) of three independently grown and treated batches of plants are shown. For abbreviations of oxylipin names, see text. ‘Oxy’ is the sum of all measured oxylipins.

## Slide 7
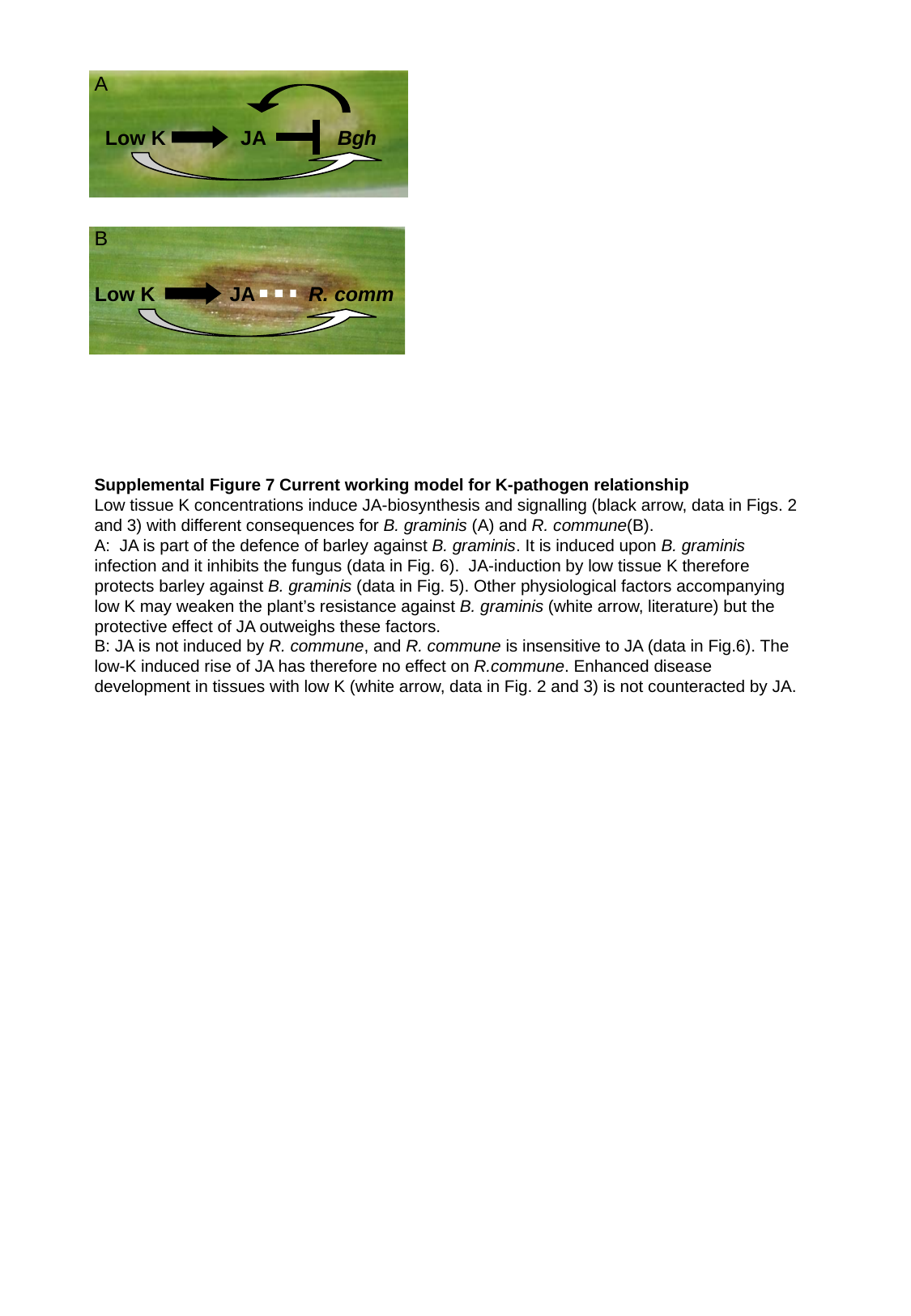

A
Low K
JA
Bgh
B
Low K
JA
R. comm
Supplemental Figure 7 Current working model for K-pathogen relationship
Low tissue K concentrations induce JA-biosynthesis and signalling (black arrow, data in Figs. 2 and 3) with different consequences for B. graminis (A) and R. commune(B).
A: JA is part of the defence of barley against B. graminis. It is induced upon B. graminis infection and it inhibits the fungus (data in Fig. 6). JA-induction by low tissue K therefore protects barley against B. graminis (data in Fig. 5). Other physiological factors accompanying low K may weaken the plant’s resistance against B. graminis (white arrow, literature) but the protective effect of JA outweighs these factors.
B: JA is not induced by R. commune, and R. commune is insensitive to JA (data in Fig.6). The low-K induced rise of JA has therefore no effect on R.commune. Enhanced disease development in tissues with low K (white arrow, data in Fig. 2 and 3) is not counteracted by JA.

## Slide 8
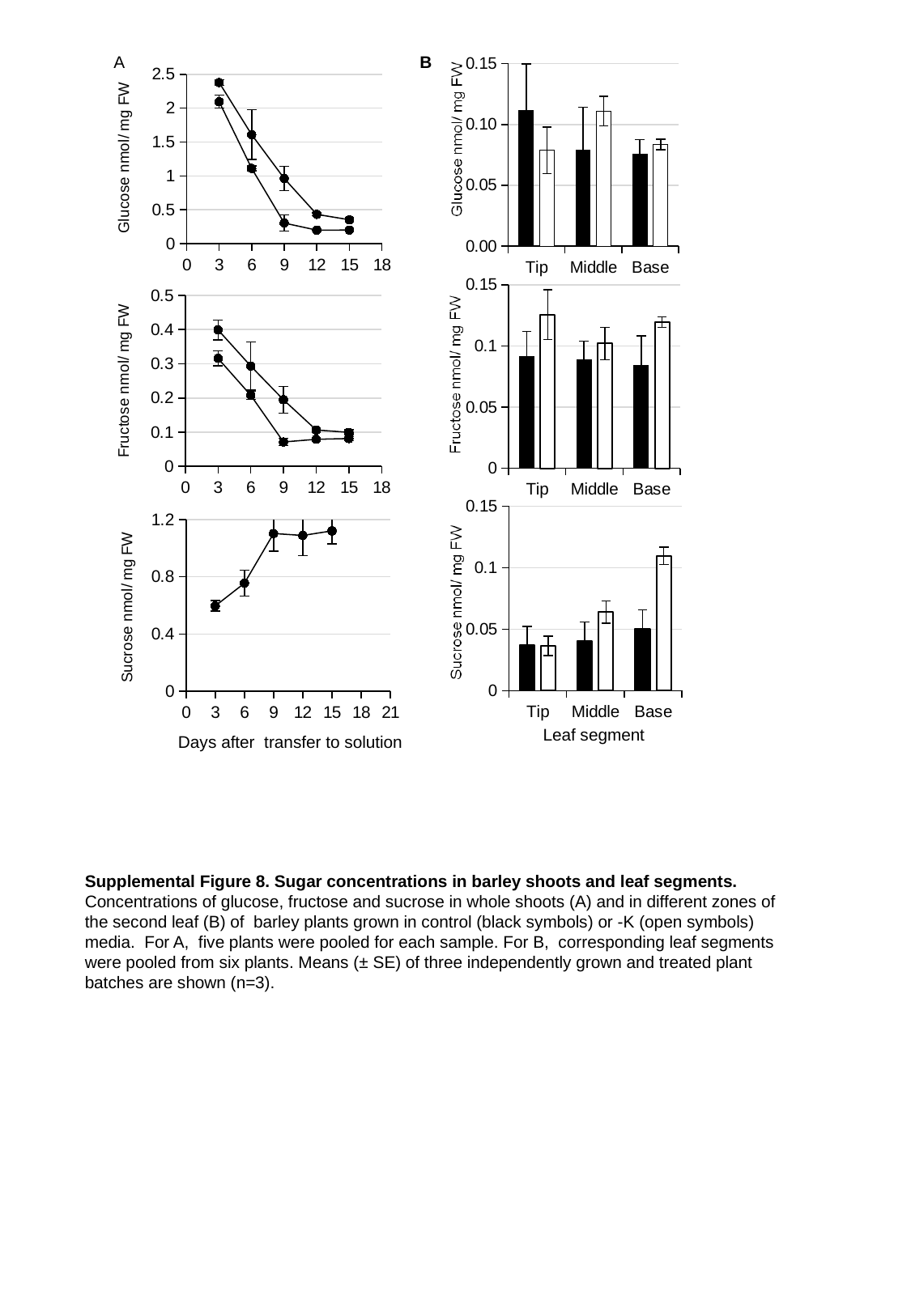

A
B
### Chart
| Category | | |
|---|---|---|
| Tip | 0.111320230058126 | 0.078872279974969 |
| Middle | 0.0790122223597925 | 0.111164989790936 |
| Base | 0.0757433757471365 | 0.0836390038441858 |
### Chart
| Category | | |
|---|---|---|
### Chart
| Category | | |
|---|---|---|
| Tip | 0.0912759144386052 | 0.125541196231757 |
| Middle | 0.0890148602288705 | 0.1020454971403 |
| Base | 0.0839886525093648 | 0.119528766615325 |
### Chart
| Category | | |
|---|---|---|
### Chart
| Category | | |
|---|---|---|
| Tip | 0.0372529646138055 | 0.0365123968725967 |
| Middle | 0.0401151948836822 | 0.0639737956723104 |
| Base | 0.0502598120285827 | 0.109590878339888 |
### Chart
| Category | | |
|---|---|---|Leaf segment
Days after transfer to solution
Supplemental Figure 8. Sugar concentrations in barley shoots and leaf segments.
Concentrations of glucose, fructose and sucrose in whole shoots (A) and in different zones of the second leaf (B) of barley plants grown in control (black symbols) or -K (open symbols) media. For A, five plants were pooled for each sample. For B, corresponding leaf segments were pooled from six plants. Means (± SE) of three independently grown and treated plant batches are shown (n=3).
